# Id2 Determines Intestinal Identity through Repression of the Foregut Transcription Factor Irx5

**DOI:** 10.1128/MCB.00250-17

**Published:** 2018-04-16

**Authors:** Kentaro Mori, Harumi Nakamura, Hisanori Kurooka, Hitoshi Miyachi, Kota Tamada, Manabu Sugai, Toru Takumi, Yoshifumi Yokota

**Affiliations:** a Division of Molecular Genetics, Department of Biochemistry and Bioinformative Sciences, School of Medicine, Faculty of Medical Sciences, University of Fukui, Eiheiji, Fukui, Japan; b Research and Education Program for Life Science, University of Fukui, Eiheiji, Fukui, Japan; c Experimental Research Center for Infectious Diseases, Institute for Virus Research, Kyoto University, Shogoin-Kawahara, Sakyo-ku, Kyoto, Japan; d RIKEN Brain Science Institute, Wako, Saitama, Japan

**Keywords:** cancer, cell differentiation, cell fate specification, endoderm, gastric mucosa, Id2, intestinal development, intestinal epithelium, Irx transcription factor, tumorigenesis

## Abstract

The cellular components and function of the gastrointestinal epithelium exhibit distinct characteristics depending on the region, e.g., stomach or intestine. How these region-specific epithelial characteristics are generated during development remains poorly understood. Here, we report on the involvement of the helix-loop-helix inhibitor Id2 in establishing the specific characteristics of the intestinal epithelium. *Id2*^−/−^ mice developed tumors in the small intestine. Histological analysis indicated that the intestinal tumors were derived from gastric metaplasia formed in the small intestine during development. Heterotopic *Id2* expression in developing gastric epithelium induced a fate change to intestinal epithelium. Gene expression analysis revealed that foregut-enriched genes encoding Irx3 and Irx5 were highly induced in the midgut of *Id2*^−/−^ embryos, and transgenic mice expressing *Irx5* in the midgut endoderm developed tumors recapitulating the characteristics of *Id2*^−/−^ mice. Altogether, our results demonstrate that Id2 plays a crucial role in the development of regional specificity in the gastrointestinal epithelium.

## INTRODUCTION

The gut tube, consisting of the endoderm and surrounding mesoderm, establishes regional identities along the rostral-caudal axis by embryonic day 8.0 (E8.0) to E9.0 and is subdivided into the foregut, midgut, and hindgut (1). Following morphological differentiation, the pseudostratified endoderm within each domain differentiates into an epithelium with a characteristic morphology and function, e.g., stomach or intestine. Ectopic epithelial tissue sometimes occurs in the gastrointestinal tract, as in Barrett's esophagus, and confers an increased risk of cancer development (2, 3). However, little is known about the mechanisms underlying the formation of ectopic epithelial tissue.

Members of the inhibitor of DNA binding/differentiation (Id) family are negative regulators of transcription factors with a basic helix-loop-helix motif. Four members of the Id family (Id1 to Id4) have been shown to play critical roles in various processes, including angiogenesis, neurogenesis, tumorigenesis, and immune development, by regulating cell differentiation (4, 5). Id proteins not only regulate cell differentiation but also stimulate G_1_/S phase transition in the cell cycle. Increased expression of Id proteins has been reported in various tumor types, including adenocarcinomas arising from the stomach and colon (6, 7). Moreover, transgenic mice expressing *Id1* in the intestinal epithelium develop intestinal tumors (8). These findings suggest that Id proteins are involved in the neoplastic process. However, despite the growth-promoting activity of Id2, *Id2*^−/−^ mice still develop tumors in the small intestine (9).

In this study, we systematically examined the small intestine in *Id2*^−/−^ mice and in transgenic mice expressing the downstream genes for *Iroquois*-related homeobox 3 and 5 (*Irx3* and *Irx5*) to determine the involvement of Id2 in establishing the specific characteristics of the intestinal epithelium. The results of this study will further our understanding of the mechanisms regulating gastrointestinal development and ectopic tissue formation.

## RESULTS

### Development of gastric tumors in *Id2^−/−^* mice.

**FIG 1:**
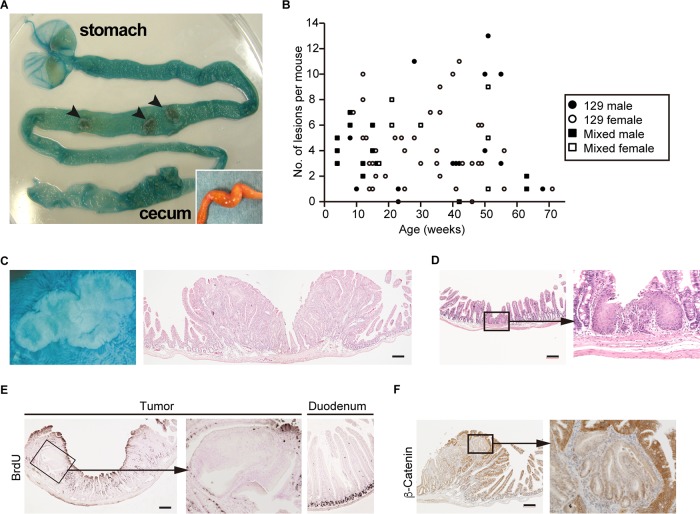
Intestinal tumors in *Id2*^−/−^ mice. (A) Gastrointestinal tract of *Id2*^−/−^ mice at 31 weeks (w) of age. The sample was opened longitudinally and stained with indigo carmine. Arrowheads indicate tumors. The inset shows intestinal distortion in the ileum of an *Id2*^−/−^ mouse. (B) Correlation between age and number of lesions in *Id2*^−/−^ mice. Genetic background and sex of mice are indicated in the box. (C) Macroscopic view (left) and histology (HE staining) (right) of a representative intestinal tumor from an *Id2*^−/−^ mouse (35 w). (D) Squamous epithelium in the small intestine of an *Id2*^−/−^ mouse (42 w). (E) BrdU-labeled tumor cells visualized by immunohistochemistry using BrdU antibody (25 w). In the normal epithelium, proliferative cells were restricted in crypts (right, duodenum). Large parts of tumor cells were low or negative for BrdU incorporation. (F) β-Catenin immunohistochemistry. High-magnification view of boxed region is shown on the right. Scale bars, 200 μm.

*Id2*^−/−^ mice developed tumors in the small intestine (9) (Fig. 1A). We found that 96% (*n* = 78/81) of *Id2*^−/−^ mice developed intestinal tumors (see Table S1 in the supplemental material). The frequency of tumor development was independent of age (Fig. 1B). Most tumors were adenomas with different grades or composed of hyperplastic epithelia and were observed in the middle to distal half of the small intestine (Fig. 1C, S1, and S2 and Table S2). The lesions had a clear glandular structure, and cytonuclear atypia was accompanied by increased tumor size (Fig. S1). Histopathological analysis of these lesions revealed that among adenomas of different grades, only an adenocarcinoma with obvious mucosal infiltration was observed. The lesions sometimes resulted in intestinal distortion accompanied by an increase in the size of the adjacent muscularis layer (Fig. 1A and C). Squamous metaplasia was also observed in the small intestine in *Id2*^−/−^ mice (*n* = 11/47) (9) (Fig. 1D). This metaplasia expressed high levels of p63 and cytokeratin 14 (CK14) and was usually localized close to a tumor (10) (Fig. S3). We further analyzed the hyperplastic tumors and adenomas. In the small intestine, the subset of proliferative epithelial cells is restricted to the crypt (11). Although tumor cells usually show high proliferative activity, bromodeoxyuridine (BrdU) labeling revealed negative or low proliferative activity in most tumor cells (Fig. 1E). Although activated Wnt signaling is commonly involved in intestinal tumor development, no β-catenin overexpression or obvious nuclear accumulation was detected by immunohistochemistry (12, 13), and β-catenin expression levels were lower in tumor cells than in adjacent normal epithelial cells (Fig. 1F).

**FIG 2:**
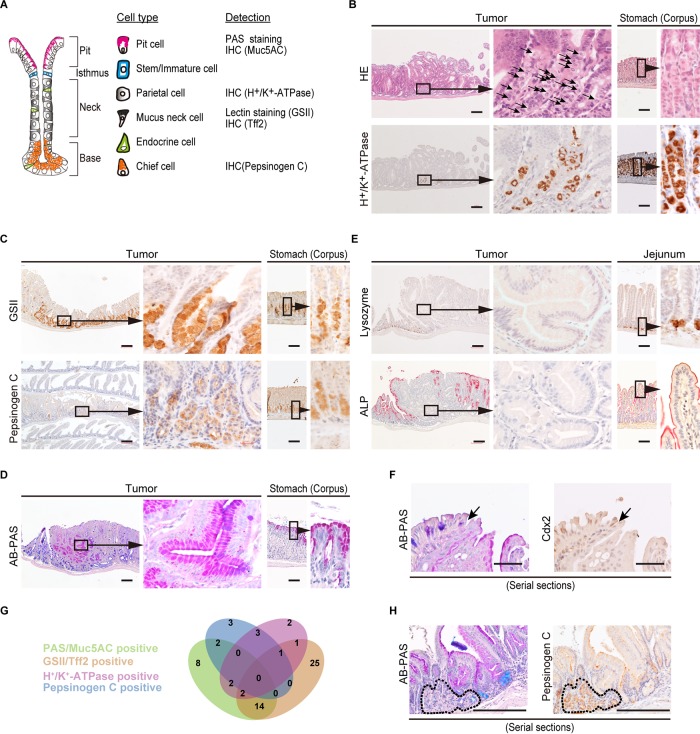
Intestinal tumors in *Id2*^−/−^ mice contained gastric cells. (A) Schematic representation of gastric unit in adult mouse. Gastric unit was subdivided into four regions characterized by the presence of specific cell types. Methods for the detection of each type of gastric cell are shown on the right. IHC, immunohistochemistry. (B, upper) Cells resembling gastric parietal cells in a tumor. (Middle) High-magnification view of boxed region. Arrows indicate cells with centrally located nuclei circumvented by intracellular canaliculus. (Lower) IHC for H^+^/K^+^-ATPase, a marker for gastric parietal cells. The panels on the right show serial sections of normal stomach epithelium stained with HE and anti-H^+^/K^+^-ATPase antibody, respectively. (C, lower) IHC using anti-pepsinogen C antibody to detect gastric chief cells in a tumor. The immunostained section represents Swiss roll of small intestine. (Upper) GSII staining showing gastric mucus neck cells in a tumor. (D) AB-PAS staining of tumor. PAS- and AB-stained gastric surface mucous cells (red) and goblet cells (blue), respectively. (E, upper) IHC using antilysozyme antibody to detect Paneth cells in a tumor. Lysozyme-positive cells are absent at the base of the intestinal tumor but present in adjacent normal intestinal crypts. (Lower) ALP staining of a tumor to detect villus columnar cells. Panels on the right show normal jejunum. (F) Epithelial boundary of the tumor. Sections of a tumor stained with AB-PAS and Cdx2, respectively. Arrows indicate transition between intestinal and gastric epithelia shown in panel D and fig. S4C. (G) Venn diagram of the results of histological analysis. Numbers indicate gastric epithelial cell marker-positive tumor. (H) Immunohistochemical analysis using serial sections. Tumor cells surrounded by a dotted line show the area where pepsinogen C-positive cells are localized. Panels D and E (lower) and Fig. S4C are serial sections. Scale bars, 200 μm.

The stomach epithelium comprises gastric units subdivided into four distinct zones based on the presence of characteristic cell types (Fig. 2A) (14). Histological examination of the tumors revealed round cells with centrally located nuclei circumvented by intracellular canaliculi, reminiscent of gastric parietal cells that secrete hydrochloride (Fig. 2B). We investigated the expression of H^+^/K^+^-ATPase, a marker for gastric parietal cells, and detected H^+^/K^+^-ATPase-positive cells in the tumors (*n* = 12/58). We also investigated other gastric gland-specific cells. Mucus neck cells, which are located in the neck region and produce acidic mucin in the gastric gland, are labeled by Griffonia simplicifolia II lectin (GSII). Tumor cells in *Id2^−/−^* mice were positive for GSII (*n* = 44/58) (Fig. 2C). These cells were also positive for another marker of mucus neck cells, Tff2 (Fig. S4A). Pepsinogen is only secreted by differentiated gastric chief cells, and immunostaining using pepsinogen II antibody showed that tumors contained gastric chief cells (*n* = 9/58). Furthermore, alcian blue–periodic acid-Schiff (AB-PAS) staining showed the presence of PAS-positive gastric surface mucus cells (pit cells) (*n* = 28/58) (Fig. 2D). These cells were also stained with Muc5AC, a marker of gastric surface mucus cells (Fig. S4B). These gastric cell types were not found in the small intestine of wild-type or *Id2*^+/−^ mice. Regions containing these gastric cell types within tumors were devoid of absorptive enterocytes, goblet cells, and Paneth cells (11) (Fig. 2D and E). Cdx2, a homeodomain-containing transcription factor and specific marker of intestinal epithelial cells, was not detected in tumor epithelium, but positive staining was detected in the adjacent intestinal epithelium (15, 16) (Fig. S4C). Cdx2-negative and -positive epithelia corresponded to those containing AB-positive differentiated goblet cells and PAS-positive gastric surface mucus cells, respectively (Fig. 2F). These findings indicate that the tumors are metaplastic changes. In Fig. 2G, we summarize the results of histological characteristics of tumors (Fig. 2G). Notably, many tumors contained either a single type or 2 or 3 types of gastric cells (Fig. 2H), and no tumors contained all types of gastric cells. Histological analysis using serial sections showed that one type of gastric cells was localized at a specific area within the tumor, while the formation of gastric glandular structures, such as gastric epithelium, was not observed. This suggests that the presence of gastric stem cells in tumors is extremely limited, and tumor cells may originate from gastric progenitor cells committed to a specific lineage. In addition, given that the anterior portion of the rodent stomach is lined with squamous epithelium, the squamous and keratinized epithelia in the small intestine in *Id2*^−/−^ mice were presumably related to foregut endoderm-derived tissue (17).

### Requirement of *Id2* for specification of intestinal identity.

**FIG 3:**
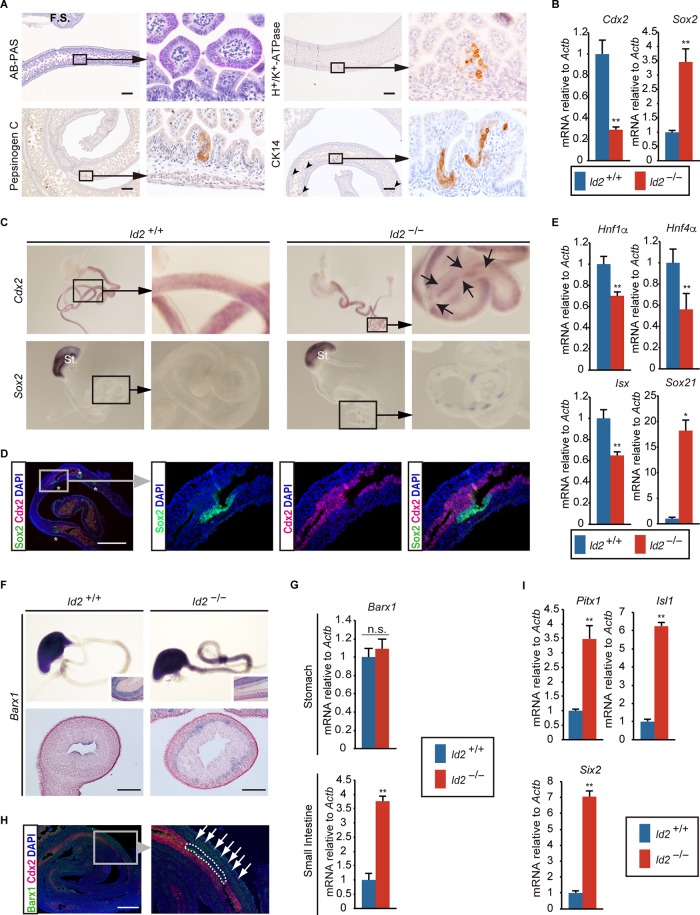
Formation of ectopic gastric epithelium in small intestine during development. (A) E18.5 *Id2*^−/−^ small intestines were stained with AB-PAS and immunostained for H^+^/K^+^-ATPase, pepsinogen C, and CK14. Arrowheads indicate CK14-positive epithelium. F.S., fetal stomach. (B) qRT-PCR analysis of *Cdx2* and *Sox2* in E13.5 *Id2*^−/−^ and wild-type (*Id2*^+/+^) midgut. Genotypes (*Id2*^+/+^ and *Id2*^−/−^) are indicated by blue-black and red, respectively (*n* = 6). Mean wild-type expression levels were set to 1. (C, upper) *Cdx2* expression. Arrows indicate local regions defective for *Cdx2* expression. (Lower) *Sox2* expression. *Sox2* was normally expressed in the developing stomach epithelium and also detected in the small intestine of *Id2*^−/−^ mice in a dot-like pattern. St., stomach. (D) Coimmunostaining for Sox2 (green) and Cdx2 (magenta) in E14.5 *Id2*^−/−^ small intestine. Panels on the left and right show merged images. Nuclei were counterstained using DAPI (blue). (E) qRT-PCR analysis of Cdx2 target genes (*Hnf1α*, *Hnf4α*, and *Isx*) and Sox2 target gene (*Sox21*) in E13.5 small intestine (*n* = 6). (F) Whole-mount ISH for *Barx1* in E14.5 gastrointestinal tract. (Upper, inset) Stomach section of embryo by each genotype shows *Barx1* expression in the mesenchyme. (Lower) Intestinal section shows ectopic *Barx1* expression in the mesenchyme. n.s., not significant. (G) qRT-PCR analysis of *Barx1* in E13.5 stomach and small intestine (*n* = 6). (H) Coimmunostaining for Barx1 (green) and Cdx2 (magenta) in E14.5 *Id2*^−/−^ small intestine. A high-magnification image of the boxed region is shown in the lower panel. Dashed circles indicate endoderm lacking Cdx2 expression. Arrows indicate mesenchyme ectopically expressing Barx1. (I) qRT-PCR analysis for *Barx1* target genes (*Pitx1*, *Isl1*, and *Six2*) in E13.5 small intestine (*n* = 6). *, *P* < 0.05; **, *P* < 0.01. Scale bars: 200 μm (A), 50 μm (D), and 100 μm (F).

We investigated the timing of heterotopic gastric epithelial formation in the small intestine in *Id2*^−/−^ mice. Gastric surface mucus cells (*n* = 18/18), parietal cells (*n* = 15/18), and chief cells (*n* = 12/18) were already identifiable by E18.5, and CK14-positive stratified squamous cells were also detected in the basal layer (*n* = 15/18) (Fig. 3A). Since tumors are confirmed early postnatally, these observations suggest that these metaplastic cells develop tumors at the early stage.

Cdx2 and Sox2 have been shown to play important roles in establishing the identity of gastric and intestinal epithelial cells, respectively. *Cdx2* deficiency causes heterotopic esophageal and/or gastric epithelial cell formation in the small intestine, while ectopic expression of *Sox2*, a foregut endoderm-enriched transcription factor, in the developing intestinal endoderm induces gastric epithelium formation (10, 18–22). We therefore examined *Cdx2* and *Sox2* expression in the developing small intestine in *Id2*^−/−^ embryos. Quantitative reverse-transcription PCR (qRT-PCR) revealed significantly reduced *Cdx2* expression in the midgut of E13.5 *Id2*^−/−^ embryos, whereas *Sox2* expression was markedly increased (Fig. 3B). We examined the *Cdx2* and *Sox2* expression patterns at E14.5 by whole-mount *in situ* hybridization (ISH). Although *Cdx2* expression was observed throughout the midgut endoderm in *Id2*^−/−^ embryos, similar to the wild type, *Cdx2*-negative regions were observed in the midgut endoderm of *Id2*^−/−^ embryos (Fig. 3C), in accordance with discontinuous *Cdx2* expression in the epithelium of intestinal tumors in *Id2*^−/−^ adult mice (Fig. 2F). *Sox2* expression was observed in the foregut endoderm in *Id2*^−/−^ embryos, similar to the wild type, while *Sox2*-positive epithelial spots were detected in the midgut of *Id2*^−/−^ mouse embryos (Fig. 3C). In addition, immunohistochemical examination of E12.5 *Id2*^−/−^ midgut revealed mutually exclusive *Sox2* and *Cdx2* expression patterns in the developing endoderm (Fig. 3D). Alterations in the expression patterns of these regionally restricted genes were associated with a decrease in Cdx2 target genes (*Hnf1α*, *Hnf4α*, and *Isx*) and an increase in Sox2 and its target gene (*Sox21*) in the developing midgut endoderm (19, 23) (Fig. 3E). We additionally examined the expression pattern of the transcription factor *Barx1*, which is expressed in the developing stomach mesenchyme and is essential for normal gastric epithelium development (24, 25). In *Id2*^−/−^ mouse embryos, *Barx1* expression levels and expression pattern in the stomach were similar to those in the wild type (Fig. 3F and G). In contrast to gastric mesenchyme-restricted expression in the wild-type gut, *Barx1* expression extended into the intestinal mesenchyme in *Id2*^−/−^ mouse embryos at E14.5, indicating that *Id2*^−/−^ mouse intestine assumed gastric characteristics (Fig. 3F and G). In contrast to the heterotopic expression patterns of *Cdx2* and *Sox2*, *Barx1* expression was not restricted to the mid-to-distal region of the developing small intestine. Immunohistochemical examination showed that *Barx1* expression in the intestinal mesenchyme was higher adjacent to the Cdx2-negative epithelium than with mesenchyme adjacent to Cdx2-positive epithelium (Fig. 3H). qRT-PCR confirmed *Barx1* expression in the developing small intestine, together with an increase in expression of its target genes (*Pitx1*, *Isl1*, and *Six2*) in *Id2*^−/−^ embryos (Fig. 3G and I) (26). In this developmental stage, Id2 expression is higher in endoderm than mesenchyme (27). To clarify whether heterotopic Barx1 expression in the intestinal mesenchyme was induced by *Id2*-deficient endoderm, we performed *in vitro* reconstitution analysis using the endoderm and mesenchyme isolated from *Id2*^−/−^ and wild-type embryos (Fig. S5A to C). RT-PCR analysis revealed that Barx1 was strongly induced in the mesenchyme cultured with *Id2*-deficient endoderm (Fig. S5D). This suggests that mesenchymal Barx1 expression is regulated by the adjacent endoderm, and that the *Id2*-deficient midgut endoderm has foregut endoderm characteristics. Furthermore, we investigated whether mesenchymal Barx1 expression is involved in the decrease in endodermal Cdx2 expression. qRT-PCR revealed no change in Cdx2 expression in the wild-type endoderm cocultured with *Id2*^−/−^ mesenchyme (Fig. S5E). These observations are consistent with the results of Jayewickreme and Shivdasani, who showed that heterotropic expression of Barx1 in the mesenchyme of the small intestine does not affect epithelial Cdx2 expression (26).

### BMP-Smad signaling is not involved in heterotopic gastric epithelial development in *Id2*^−/−^ mice.

Bone morphogenetic protein (BMP) signaling is known as one of the most important mechanisms in regionally restricted gastrointestinal epithelium development, and Id family members are typical BMP targets in various cell types (28–31). We investigated whether *Id2* deficiency affects BMP-Smad signaling in the developing gastrointestinal tract. qRT-PCR analysis revealed no obvious change in the expression of BMP-Smad signaling components, which were highly expressed in the developing endoderm (Fig. S6A) (29, 32). Western blotting indicated that phosphorylated Smad1/5/8 levels in the *Id2*^−/−^ midgut were nearly the same as those in the wild type (Fig. S6B). Furthermore, immunohistochemistry analysis showed that phosphorylated Smad-1/5/8 levels in the Cdx2-negative endoderm were similar to those in the adjacent Cdx2-positive endoderm (Fig. S6C). These results suggest that Id2 deficiency does not affect BMP-Smad signaling. Id1, a member of the Id family, is also a target of BMP-Smad signaling and is known to be expressed in the endoderm (8, 29). Interestingly, the expression of Id1 and other BMP-Smad signaling targets (*Msx1* and *Msx2*) was significantly increased in the *Id2*^−/−^ midgut (Fig. S6B and C). These results suggest the presence of another transcriptional regulatory mechanism that compensates for *Id2* deficiency in the endoderm, and Id1 may function to establish intestinal identity similar to that of Id2.

### Wnt signaling is attenuated in *Id2*^−/−^ mice in the late stage of development of small intestine.

**FIG 4:**
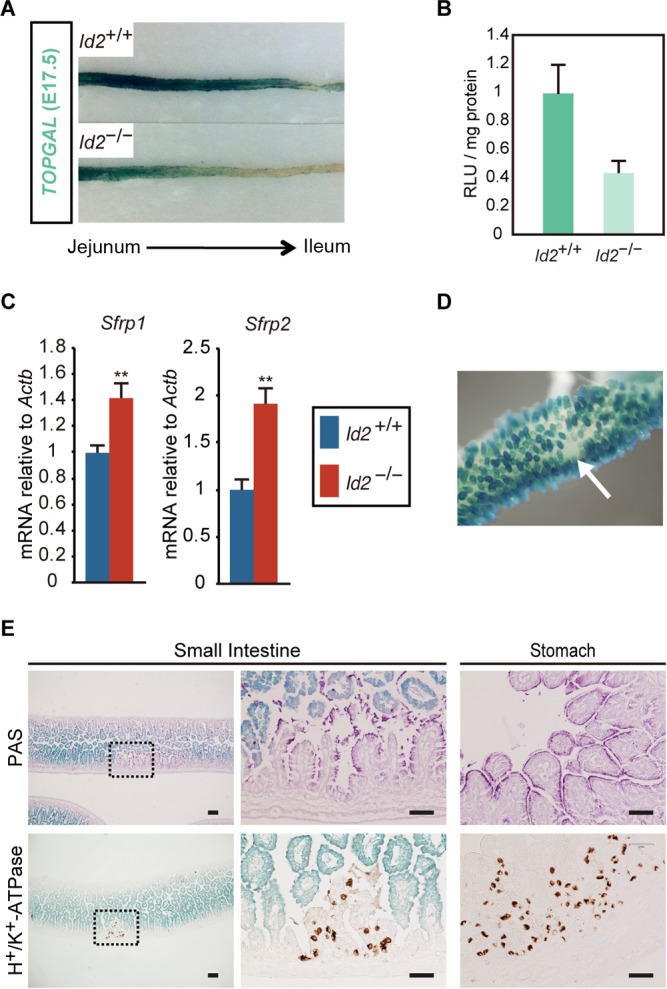
Wnt signaling is attenuated in the developing *Id2*^−/−^ small intestine. (A) β-Galactosidase staining of small intestine from E17.5 *Id2*^+/+^ and *Id2*^−/−^ embryo harboring TOP-GAL reporter (*Id2*/TOP-GAL). Tissues were dissected longitudinally, and β-galactosidase (β-gal) activity was detected. The image represents epithelial surface of small intestine between the jejunum and ileum. (B) Quantification of β-gal activities of small intestine *Id2*/TOP-GAL embryo. RLU, relative light units. (C) qRT-PCR analysis of *Sfrp1* and *Sfrp2* expression in E16.5 small intestine of *Id2*^−/−^ embryo (*n* = 5). (D) The spot disappearance of β-gal activity in E17.5 small intestine of *Id2*^−/−^/TOP-GAL embryo. The arrowhead indicates an epithelial region lacking β-gal activity (*n* = 4). (E) Histological analysis of E18.5 *Id2*^−/−^/TOP-GAL small intestine. (Upper, left) PAS staining. (Upper, middle) High-magnification image of boxed region shown on the left. (Upper, right) PAS staining of E18.5 *Id2*^−/−^/TOP-GAL stomach tissue. (Lower, left) IHC for H^+^/K^+^-ATPase. (Lower, middle) High-magnification image of boxed region shown on the left. (Lower, right) H^+^/K^+^-ATPase of E18.5 *Id2*^−/−^/TOP-GAL stomach tissue. **, *P* < 0.01. Scale bars, 50 μm.

During development of the gastrointestinal tract, it is well established that spatiotemporal Wnt signaling activities play essential roles in regionally specific epithelial development (24, 33, 34). In the midgut region, Wnt signaling was observed prior to gut tube formation and after the intestinal epithelial fate specification stage. In the developing stomach, Barx1 inhibits Wnt signaling by inducing the Wnt antagonists Sfrp1 and Sfrp2 during stomach epithelial cell differentiation (24). In the developing small intestine, canonical Wnt signaling activity is observed in the epithelium of premature intestinal villi after E16 (24, 34). To examine whether Wnt signaling is attenuated in the developing small intestine of *Id2*^−/−^, we examined Wnt signaling in TOP-GAL reporter mice, which express Escherichia coli β-galactosidase (LacZ) under the control of Tcf/Lef-responsive DNA sequences (34, 35). Remarkably, TOP-GAL reporter activity was significantly decreased in the mid-to-distal region of *Id2*^−/−^ mouse embryos (Fig. 4A and B). At this stage, *Sfrp1* and *Sfrp2* expression levels were significantly higher in the small intestine of *Id2*^−/−^ mice than in wild-type mice (Fig. 4C). In addition to widespread reduction in Wnt signaling, regions where TOP-GAL reporter activity was completely lacking were detected (Fig. 4D). These regions were positive for gastric epithelial cell markers (Fig. 4E). These results indicate that *Id2* deficiency causes widespread reduction of Wnt signaling after midgestation and support the hypothesis that suppression of Wnt signaling is required for stomach epithelial development (24).

Overall, these findings suggest that Id2 is involved in establishing intestinal identity during embryogenesis, probably through epithelial-mesenchymal interactions that are essential for proper organ development (1, 17, 19, 24).

### Effect of ectopic *Id2* on fate of foregut endoderm.

**FIG 5:**
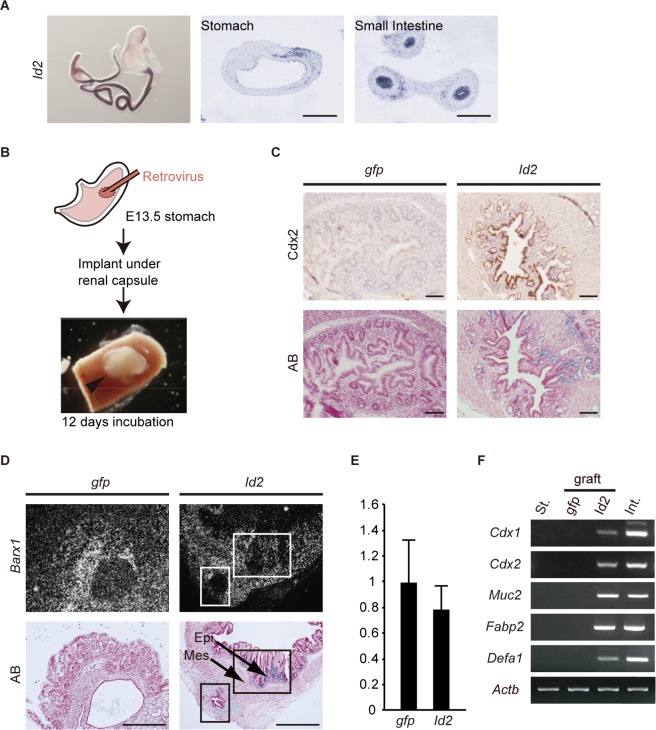
Ectopic *Id2* expression induced intestinal epithelial cells in the developing stomach. (A) Id2 expression in the developing gastrointestinal tract. Whole-mount ISH (left) and ISH of sections (middle and right) for *Id2* at E12.5. (B) Experimental strategy for ectopic *Id2* expression in embryonic stomach. The arrowhead indicates the recovered stomach. (C) Immunohistochemistry of recovered stomachs. Left and right panels are serial sections. Transduced genes are indicated at the top. Induced intestinal epithelial cells were positive for Cdx2 (brown). AB stained goblet cells blue. (D) ISH of recovered stomachs. Upper and lower panels show dark-field views of ISH and AB staining, respectively. Transduced genes are indicated at the top. Boxes indicate regions in which *Barx1* was absent from the mesenchyme adjacent to the epithelium containing AB-positive goblet cells. Epi, epithelium; Mes, mesenchyme. (E) qRT-PCR analysis for Barx1 in graft (*n* = 4). (F) RT-PCR analysis of recovered stomachs. St., wild-type stomach at E18.5; *gfp*, stomach transduced with EGFP cDNA; *Id2*, stomach transduced with *Id2* cDNA; Int., wild-type small intestine at E18.5; graft, engrafted tissues. *Actb* served as an internal control. Scale bars: 50 μm (A) and 100 μm (C and D).

During development, *Id2* expression was high in the intestinal epithelium but barely detectable in the stomach (Fig. 5A) (27). To examine the effect of ectopic *Id2* expression in the developing gastric epithelium, we retrovirally transduced *Id2* into E13.5 stomach epithelium and allowed its further development under the renal capsule in syngeneic mice (Fig. 5B). Immunohistochemical and histochemical analyses revealed that engrafted *Id2*-transduced tissues contained epithelial cells that were positive for Cdx2 expression and AB staining (*n* = 14/26) (Fig. 5C), suggesting that *Id2* expression induced intestinal differentiation in the gastric epithelium. In addition, ISH demonstrated that *Barx1* expression was absent from the mesenchyme surrounding the heterotopic intestinal epithelium, although other regions of the engrafted stomach mesenchyme retained *Barx1* expression (Fig. 5D). Intestinal epithelial cells and *Barx1*-negative mesenchymal regions were not observed in control grafts. qRT-PCR analysis confirmed the reduction of *Barx1* expression in the Id2-expressing stomach (Fig. 5E). Consistent with these findings, RT-PCR revealed that intestine-specific transcripts, including *Cdx1*, *Cdx2*, *Muc2*, *Fabp2*, and *Defa1*, were expressed in *Id2*-transduced grafts but not in controls (Fig. 5F). In the developing stomach, the foregut mesenchyme expresses Id2 (Fig. 5A) (27). To assess the role of mesenchymal Id2 in stomach epithelial cell fate conversion, we retrovirally transduced *Id2* into E13.5 *Id2*^−/−^ stomach epithelium (Fig. S7A). Immunohistochemical and histochemical analyses revealed that the engrafted *Id2*-transduced *Id2*^−/−^ stomach contained epithelial cells that were positive for Cdx2 expression and AB staining (*n* = 3/5) (Fig. S7B). qRT-PCR analysis revealed reduced *Barx1* expression in the Id2-expressing *Id2*^−/−^ stomach (Fig. S7C). Furthermore, RT-PCR analysis revealed that intestine-specific transcripts were also expressed in *Id2*-transduced *Id2*^−/−^ grafts (Fig. S7D).

Altogether, these results indicate that Id2 converted the fate of the developing stomach to that of the intestine, in both the epithelium and adjacent mesenchyme, and suggest that loss of epithelial *Id2* expression is required for stomach-specific development.

### Role of *Id2* in repression of foregut gene expression in the midgut.

**FIG 6:**
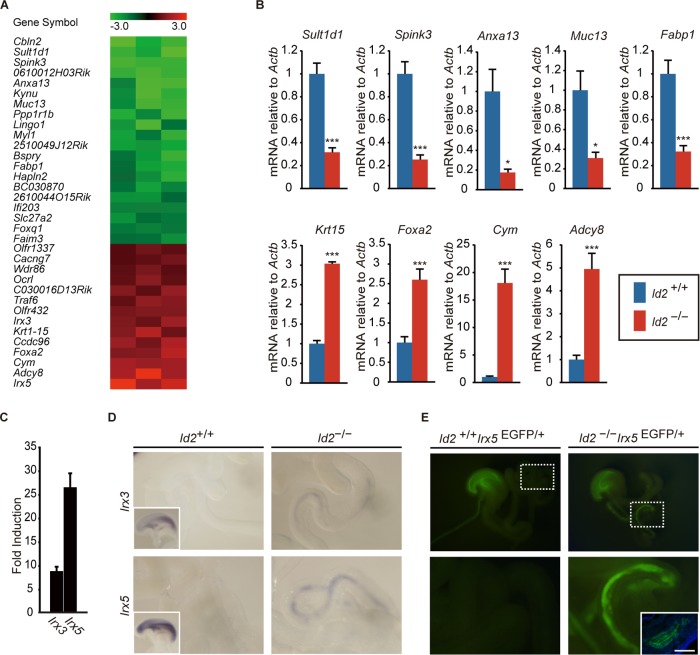
Gene expression in developing small intestine of *Id2*^−/−^ embryos. (A) Heatmap of genes showing at least 2-fold differential expression in the distal half of the small intestine in *Id2* wild-type (*Id2*^+/+^) and *Id2*-deficient (*Id2*^−/−^) mouse embryos at E13.5 (*n* = 3). Fold changes (*Id2*^+/+^/*Id2*^−/−^) of normalized signal values were converted into log_2_ ratios. The color scale at the top of the heatmap is log based. (B) qRT-PCR analysis of gene expression in E13.5 intestine. Genes known to exhibit region-dependent expression were examined and compared between *Id2*^+/+^ and *Id2*^−/−^ mice. Midgut endoderm-enriched genes (*Sult1d1*, *Spink3*, *Anxa13*, *Muc13*, and *Fabp1*) were markedly decreased in *Id2*^−/−^ embryonic intestines. In contrast, genes normally expressed in the foregut endoderm (*Krt15*, *Foxa2*, *Cym*, and *Adcy8*) were increased in *Id2*^−/−^ embryonic intestines. *, *P* < 0.05; ***, *P* < 0.005; *n* = 6 per genotype. (C) qRT-PCR analysis of *Irx3* and *Irx5* in E13.5 small intestine. Expression levels in *Id2*^−/−^ mice compared with *Id2*^+/+^ mice are shown as fold induction (*n* = 6 per genotype). Error bars show SEM. (D) Whole-mount ISH for *Irx3* and *Irx5* in E14.5 small intestine. Insets in the left panel show esophagus and stomach regions. (E) EGFP expression in the gastrointestinal tract of E14.5 *Id2*^−/−^ *Irx5*^EGFP/+^ embryo. High-magnification views of the dashed boxed regions are shown in the respective lower panels. The inset shows immunostaining for EGFP. Scale bar, 50 μm.

To understand the mechanisms underlying Id2-mediated fate control of the developing digestive tract, we analyzed gene expression using microarrays in the small intestine of E13.5 *Id2*^−/−^ embryos (Fig. 6A and Table S3). Expression levels of several foregut/midgut-enriched genes were altered in *Id2*^−/−^ embryos (Fig. 6B). Genes that varied in *Id2*^−/−^ embryos also contained genes highly expressed in the pancreas and liver. In Id2 mice, however, there was no significant change in the histology of the pancreas or liver. We focused on *Irx3* and *Irx5*, members of the *Iroquois* homeobox gene family, which are known to be involved in organ patterning and specification. Their expression is normally restricted to the foregut endoderm in the developing gastrointestinal tract (36–38). qRT-PCR revealed that *Irx3* and *Irx5* expression levels were 8.7- and 26.4-fold higher, respectively, in E13.5 *Id2*^−/−^ intestine than in controls (Fig. 6C). Although *Irx3* and *Irx5* were restricted to the foregut epithelium in wild-type embryos, whole-mount ISH at E14.5 confirmed ectopic expression in the small intestine of *Id2*^−/−^ embryos (Fig. 6D). Furthermore, *Id2*^−/−^ mice crossed with *Irx5*^+/EGFP^ reporter mice, which express enhanced green fluorescent protein (EGFP) under the control of the *Irx5* promoter, showed ectopic EGFP expression in the midgut endoderm (Fig. 6E). These results suggest that Id2 suppresses foregut-restricted gene expression during small intestine development.

### Gastric tumor development in the small intestine in *Irx5* transgenic mice.

**FIG 7:**
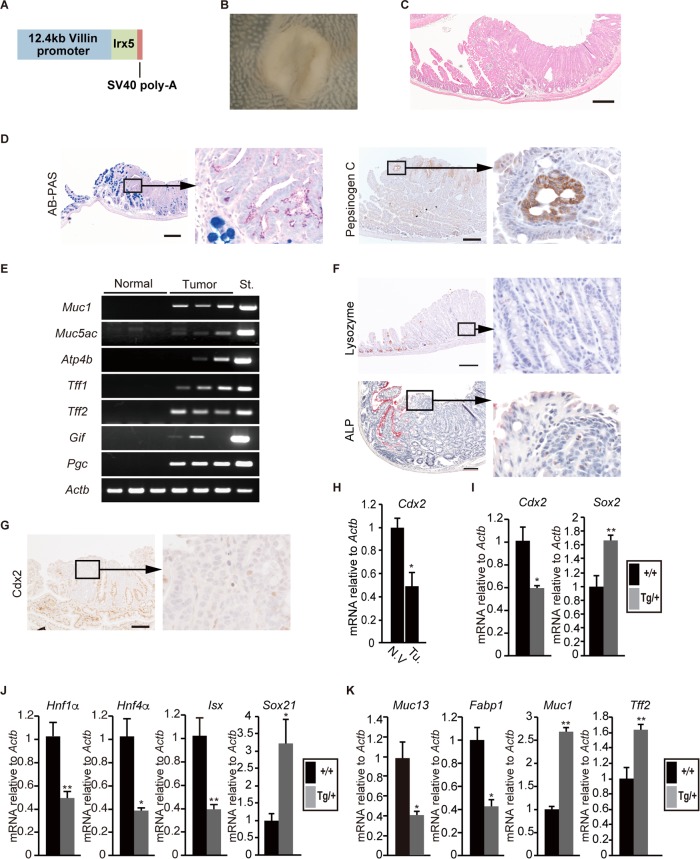
Heterotopic *Irx5* expression leads to development of gastric tissue in the small intestine in *Irx5*-Tg mice. (A) Schematic of transgene construct, consisting of mouse *Irx5* coding region with 12.4 kb of the mouse villin promoter at the 5′ region and simian virus 40 (SV40) poly(A) recognition sequences at the 3′ region. (B) Macroscopic view of representative intestinal tumor in *Irx5*-Tg mouse (62 w). (C) Histology of tumor shown in panel B. (D, left) AB-PAS staining of tumor (53 w). (Right) Immunostaining using anti-pepsinogen C antibody to detect gastric chief cells (52 w). High-magnification views of boxed regions of the respective panels on the left. (E) RT-PCR showed expression of gastric epithelial cell-specific genes at various levels. Lanes 1 to 5 (from left), normal small intestine; lanes 6 to 10, tumors. St., wild-type stomach of 6-week-old mice. *Actb* served as an internal control. (F, upper) Immunostaining using antilysozyme antibody to detect Paneth cells. Positive signal (brown) missing at the tumor base. (Lower) ALP staining. Apical surface of the intestinal villi stained positive (red), while the tumor was negative. (G) Cdx2 immunostaining (52 w). High-magnification view of boxed region shown on the right. (H) qRT-PCR analysis of *Cdx2* expression in normal villi and small intestinal tumors in *Irx5*-Tg mice. N.V, normal villi; Tu., tumor. (I) *Cdx2* and *Sox2* expression in E18.5 *Irx5*-Tg (Tg/+) small intestine compared with wild-type (+/+) intestine. (J) qRT-PCR analysis of Cdx2 target genes (*Hnf1α*, *Hnf4α*, and *Isx*) and Sox2 target gene (*Sox21*) in E18.5 small intestine (*n* = 5). (K) Intestine-specific genes (*Muc13* and *Fabp1*) were significantly downregulated, while stomach-specific genes (*Sox21*, *Muc1*, and *Tff2*) were upregulated. *, *P* < 0.05; **, *P* < 0.01; *n* = 5. Scale bars, 200 μm.

To determine if the Irx transcription factor is involved in ectopic gastric epithelial cell development, we generated transgenic mice expressing *Irx5* cDNA (*Irx5*-Tg) under the control of the villin promoter (39) (Fig. 7A). Immunohistochemistry and qRT-PCR confirmed Irx5 expression in the developing small intestine, with expression levels comparable to those of *Id2*^−/−^ embryos (23.3-fold) (Fig. S5). We found that 16% (*n* = 11/68) of *Irx5*-Tg mice older than 50 weeks developed a total of 13 intestinal tumors (Fig. 7B and C and Tables S4 and S5). Immunohistochemistry showed that the tumors contained PAS-positive gastric surface mucus cells (*n* = 8/13) and pepsinogen C-positive gastric chief cells (*n* = 4/13) (Fig. 7D). RT-PCR analysis revealed that the tumors expressed gastric pit cell-specific (*Muc1*, *Muc5ac*, and *Tff1*), mucus neck cell-specific (*Tff2*), parietal cell-specific (*Atp4b*), and chief cell-specific (*Gif* and *Pgc*) transcripts at various levels (Fig. 7E). However, we did not observe ectopic stratified squamous epithelium in the small intestine of *Irx5*-Tg mice. Histological examination also indicated that all tumors were devoid of goblet cells, Paneth cells, and alkaline phosphatase (ALP)-positive absorptive enterocytes (Fig. 7D and F). In addition, a region of the tumor showed obvious reduction or complete loss of Cdx2 staining (Fig. 7G). Alteration of *Cdx2* expression was confirmed by qRT-PCR (Fig. 7H). These results suggest that ectopic *Irx5* expression induced the formation of ectopic gastric epithelial tissue in the mouse small intestine. To determine if heterotopic *Irx5* expression affected regionally restricted gene expression, we analyzed *Cdx2* and *Sox2* expression levels in the developing small intestine. At E18.5, *Cdx2* expression was markedly reduced in *Irx5*-Tg mouse embryos, while *Sox2* expression was significantly increased (Fig. 7I). Furthermore, heterotopic *Irx5* also downregulated Cdx2 targets (*Hnf1α*, *Hnf4α*, and *Isx*) and intestinal epithelium-specific transcripts (*Muc13* and *Fabp1*) and upregulated an Sox2 target (*Sox21*) and stomach epithelium-specific transcripts (*Muc1* and *Tff2*) (Fig. 7J and K). These results suggest that heterotopic *Irx5* expression affected the cellular identity of the small intestinal epithelium and caused some of the endoderm to adopt a stomach epithelium cell fate. In addition, *Cdx2* downregulation may destabilize intestinal identity (18–20).

## DISCUSSION

Previous studies have shown that various factors play important roles in gastrointestinal tract patterning. However, despite widespread analysis focused on the role of specific transcriptional factors, the regulatory mechanisms involved remain elusive. Based on the analysis of a transcriptional regulator, we previously elucidated the mechanisms of cell fate specification underlying tissue-specific differentiation (5, 40–42). In the present study, we showed that Id2 was involved in the cell fate determination of small intestine epithelial cells through the repression of foregut differentiation programs during development.

*Id2*^−/−^ mice developed heterotopic squamous epithelium and gastric tumors containing all types of gastric cells in the small intestine. In the current study, most gastric tumors were adenomas or hyperplastic epithelium, and heterotopic gastric cells were identified in the embryonic small intestine.

Nearly all tumors had well-differentiated gastric cells, and the cytonuclear atypia appeared to be increased with increasing tumor size. Furthermore, tumor cells had a clear glandular form in tumors of all sizes. These are characteristics of tumors with low malignancy. Tumor carcinogenesis involves a process of adenoma formation (12, 13). *APC* mutation occurs in most human colorectal cancers. We recently reported that Id2 is involved in the tumor initiation process, and Id2 deficiency does not promote carcinogenesis of intestinal adenoma induced by *Apc* gene mutation (43). In addition, we observed no carcinoma development until 20 weeks of age. Thus, the carcinogenesis of intestinal tumors of *Id2*^−/−^ mice was likely induced by another mechanism, such as secondary gene mutation.

Given that the frequency of tumor development was age independent and *Id2* expression was barely detectable in intestinal epithelial cells after birth, intestinal tumors in *Id2*^−/−^ mice appeared to be derived from ectopic gastric cells formed in the small intestine during development. Immune responses are recognized as important factors in tumor formation, including gastrointestinal cancer (44). We previously reported that *Id2*^−/−^ mice lacked secondary lymphoid organs, including lymph nodes and Peyer's patches (40). In addition, *Id2* deficiency caused a defect in natural killer cell differentiation and a significant reduction in the number of intestinal epithelial lymphocytes (45). The lack of Peyer's patches and impaired natural killer cells and intestinal epithelial lymphocytes, which play crucial roles in immunosurveillance against tumor development, may have an influence on tumor formation in the small intestine of *Id2*^−/−^ mice. Notably, the number of ectopic gastric cells in postnatal mice was reduced compared with that in embryos. We previously reported that litter sizes of *Id2* mutant mice were similar to those of wild-type mice, with no apparent increase in embryonic lethality; however, approximately 25% of *Id2*^−/−^ newborn mice died in the neonatal period (40). Most neonatal-lethal mice exhibited severe intestinal distortion, indicating that mortality in *Id2*^−/−^ mice during the neonatal period was associated with the frequency of ectopic gastric cells.

*Id2* is expressed in the developing midgut endoderm from E9.0. High levels of *Id2* expression continue until around E14.5, but then they decrease gradually in parallel with morphological differentiation from pseudostratified endoderm to intestinal villi (27). During this period, the midgut endoderm shows considerable plasticity. Conditional ablation of *Cdx2* in the early definitive endoderm resulted in replacement of the posterior intestinal epithelium with keratinized squamous epithelium, while ablation at E13.5 led to the formation of ectopic gastric tissue in the small intestine without keratinized squamous epithelium (19, 20). These observations indicate that *Cdx2* directs the endoderm toward a posterior gut phenotype, and that loss of *Cdx2* impacts differentially on intestinal patterning in a temporal manner. However, ectopic *Sox2* expression throughout the midgut endoderm from E8.5 directs development toward gastric properties (21). Our findings of Sox2-positive and Cdx2-negative cells in the *Id2*-deficient endoderm suggest that Id2 sustains cellular identity by regulating both of these genes prior to the initiation of intestinal epithelial cell differentiation.

*Barx1*-deficient mice showed defects in stomach development (24, 25). In these mice, the stomach epithelium was completely replaced by Cdx2-positive endoderm. Although Barx1 is thought to suppress endoderm Cdx2 expression, heterotopic Barx1 expression in the mesenchyme of the small intestine did not change endodermal Cdx2 expression (26). We observed that the absence of Cdx2 expression is local, whereas Barx1 expression spans a wide spread in the midgut, indicating that Cdx2 deficiency is caused by a cell-autonomous mechanism but not induced by Barx1. The regulatory mechanisms of Barx1 expression in the stomach mesenchyme are unclear. Our *in vitro* reconstitution model using *Id2*^−/−^ mouse embryos may be useful for clarifying these mechanisms.

Transcription factors in the basic helix-loop-helix (bHLH) family play essential roles in various cell fate determination and differentiation processes (4). Tissue-specific bHLH factors form dimers with ubiquitously expressed bHLH factors known as E proteins (e.g., E2A gene products, E2-2, and HEB) and regulate tissue-specific gene expression that promotes cellular differentiation. Id proteins directly bind E proteins, resulting in inhibition of transcriptional activity of tissue-specific bHLH factors. Id proteins are negative regulators of bHLH factors; therefore, some bHLH factors may be involved in the specification of stomach epithelial cell fate. Furthermore, the transcriptional activity of such bHLH factors would be upregulated in the undifferentiated midgut endoderm of *Id2*^−/−^ mouse embryos, and the functional dysregulation resulting from Id2 deficiency may cause ectopic tissue formation. Indeed, premature neural differentiation is observed in *Id1*^−/−^ *Id3*^−/−^ mice, in accordance with upregulation of neural cell-specific bHLH expression and other markers of neural differentiation (46). Our data show that Id2 induces the fate conversion of the stomach epithelium to intestinal epithelium and suggests that ectopic Id2 represses the activity of certain bHLH factors required for stomach epithelial cell differentiation (Fig. 5; see also Fig. S7 in the supplemental material). It was demonstrated that several bHLH factors are involved in gastrointestinal epithelial cell differentiation (47). Neurogenin3 is a bHLH factor expressed in the developing gastrointestinal tract and is essential for enteroendocrine cell differentiation (48, 49). Interestingly, while the small intestine of neurogenin3-deficient mice lacks all lineages of enteroendocrine cells, the stomach epithelium exhibited an intestinal phenotype without obvious morphological changes (49). This observation suggests that neurogenin3 regulates both enteroendocrine differentiation and region-specific epithelial cell specification. However, overexpression of neurogenin3 throughout the developing intestinal epithelium did not induce fate conversion from the intestinal epithelium to the stomach epithelium. Furthermore, if neurogenin3 is a regulatory target of Id2, functional upregulation of neurogenin3 is expected in *Id2*^−/−^ mice; therefore, accelerated endocrine cell differentiation may occur. However, the numbers or subsets of endocrine cells in *Id2*^−/−^ mice were similar to those in wild-type mice (50) (K. Mori, unpublished data). The bHLH factors expressed throughout the undifferentiated endoderm that direct development toward the gastric epithelium have not been identified. Id2 may participate as a negative regulator of unidentified bHLH factors and function in establishing intestinal identity.

Gene expression analysis revealed that the *Irx* genes *Irx3* and *Irx5* were expressed ectopically in the midgut of *Id2*^−/−^ embryos. Irx transcription factors are found in multiple organisms and have been implicated in patterning and specification of several organs (51–54). Genome-wide association studies revealed that *IRX3* and *IRX5* expression relied on a long-range *cis*-regulatory element (55–57). Assuming that Id proteins are negative transcriptional regulators, Id2 may be involved in the regulation of such a *cis*-acting element, responsible for foregut endoderm-specific expression. Because the *Id2*-deficient midgut endoderm showed properties of both anterior and posterior endoderm, epigenetic analysis of midgut endoderm from *Id2*^−/−^ embryos might help to identify the functional genomic region responsible for foregut endoderm-specific *Irx* gene expression.

We also demonstrated that *Irx5*-Tg mice developed intestinal tumors that recapitulated some of the characteristics of ectopic epithelia found in *Id2*^−/−^ mice. *Cdx2* expression in the developing small intestine in *Irx5*-Tg embryos was downregulated to approximately half that of control embryos, whereas *Sox2* expression was significantly upregulated. *Cdx2* heterozygote mice have been reported to develop colonic polyps containing gastric tissues, including squamous epithelium and a gastric gland, and *Cdx2* haploinsufficiency in the developing small intestine was shown to result in fate conversion from a midgut to foregut endoderm phenotype (18). Our results suggest that Irx5 directs the undifferentiated midgut endoderm to a foregut phenotype.

*Sox2* expression and ectopic gastric tissues in the small intestine of *Id2*^−/−^ mice were restricted to spot-like regions. However, ectopic *Irx5* expression was detected over a broader area than *Sox2*. This inconsistency between the regionally restricted formation of ectopic tissues and the altered *Irx5* expression pattern implies that other genes also are involved in determining gastric cell fate. Bonnard et al. reported that Irx5 required direct interacting partners, including Irx3, to modulate transcriptional regulation (58). These observations suggest that the combined action of both factors is necessary for epithelial cell fate specification.

In conclusion, we demonstrated that *Id2* regulates intestinal fate specification by inhibiting the foregut differentiation program. Further studies to determine how *Id2* regulates *Irx* expression in the midgut endoderm, and if both *Irx3* and *Irx5* are involved in the fate specification of endodermal cells, will provide further insights into the molecular mechanisms underlying gastrointestinal organ development and ectopic epithelial tissue formation.

## MATERIALS AND METHODS

### Mice.

*Id2* mutant mice on 129/Sv or mixed (129/Sv × NMRI) genetic backgrounds were used for these studies (40). TOP-GAL reporter mice harboring the *Id2*^−/−^ genetic background were maintained on 129/Sv mice. *Id2*^−/−^ *Irx5*^EGFP/+^ compound mutant mice were generated by crossing *Id2*^+/−^ *Irx5*^EGFP/+^ mice maintained on a 129/Sv genetic background. The *Irx5* transgene construct was generated by cloning mouse *Irx5* cDNA into the SmaI/KpnI sites of a villin promoter-driven expression plasmid, 12.4-kb villin ΔATG (plasmid 19358; Addgene, Cambridge, MA). All mice were maintained under specific-pathogen-free conditions, and all experimental procedures followed the guidelines of the University of Fukui for animal experiments.

### Histological and immunohistochemical analyses.

Tissues were fixed with 4% paraformaldehyde and paraffin embedded using standard methods. Hematoxylin and eosin (HE), PAS, AB, and AB-PAS staining were performed according to standard methods (59). BrdU labeling and detection were performed using a cell proliferation kit (GE Healthcare, Milwaukee, WI). *Id2*^−/−^ mice were injected intraperitoneally with labeling solution provided with the kit (100 μl/g body weight). At 2 h postinjection, tumors and intestinal tissues were collected and fixed in 4% paraformaldehyde–phosphate-buffered saline. ALP staining was performed using a Vector red alkaline phosphatase substrate kit I (Vector Laboratories, Burlingame, CA). Gastric mucus neck cells were detected with 2 μg/ml biotin-labeled GSII (Vector Laboratories) (60), and lectin binding was visualized using a Vectastain ABC kit (Vector Laboratories). Immunofluorescent staining samples were mounted with Vectashield reagent containing DAPI (4′,6-diamidino-2-phenylindole, dihydrochloride) (Vector Laboratories). The antibodies used for immunostaining are listed in Table S6 in the supplemental material.

### *In situ* hybridization.

Whole-mount ISH using digoxigenin-labeled probes and ISH using a [^35^S]CTP-radiolabeled riboprobe were performed as described previously (40, 41). The following cDNAs were used for ISH studies: *Cdx2* cDNA (NM_007673; nucleotide [nt] 412 to 1016), *Sox2* cDNA (NM_011443; nt 487 to 1199), *Id2* (NM_010496; nt 71 to 769), *Barx1* (NM_007526; nt 472 to 1212), *Irx3* (NM_008393; nt 1186 to 1986), and *Irx5* (NM_018826; nt 1415 to 1987).

### β-Galactosidase staining and quantitative assay.

*β*-Galactosidase staining was performed according to the method of Kim et al. (24). For quantification of β-galactosidase activity, the middle one-third of small intestine segments were dissected, homogenized, and lysed in 200 μl of reporter lysis buffer with a β-galactosidase enzyme assay system (Promega, Madison, WI). The relative enzyme activity was determined by reading the absorbance of the samples at 420 nm with a Spectra EMax microplate reader (Molecular Devices, CA). β-Galactosidase activity was normalized against protein concentration for each sample.

### Ectopic *Id2* expression in embryonic stomach.

Retroviruses were produced by transfecting pMX-IRES-Id2 or pMX-IRES-GFP plasmids into Plat-E packaging cells as described previously (42, 61). Embryonic stomach (E13.5) was infected with retroviruses in Dulbecco's modified Eagle's medium with 8 μg/ml Polybrene, incubated at 37°C in a 5% CO_2_ humidified atmosphere for 2 h, and then transplanted under the renal capsule of a syngeneic 8-week-old male mouse. Transplanted tissue was recovered and analyzed after 12 days.

### RT-PCR.

Total RNA samples were extracted using an RNeasy minikit (Qiagen, Valencia, CA), and oligo(dT)-primed first-strand cDNAs were synthesized using SuperScript III reverse transcriptase (Invitrogen, Carlsbad, CA). qRT-PCR was performed with each primer set using Power SYBR green PCR master mix and a StepOnePlus real-time PCR system (Applied Biosystems, Foster City, CA). Data were normalized relative to *Actb* amplification. The PCR primers are listed in Table S7. The PCR conditions are available on request.

### Microarray analysis.

Total RNA samples were extracted from the distal half of E13.5 midgut tissue. One microgram of total RNA was amplified for one round using a NanoAmp RT-IVT labeling kit (Applied Biosystems). Microarray analysis was performed using mouse genome survey microarray ver.2.0 (Applied Biosystems) (62). Raw signal values were normalized by the median. In all experiments, probe sets with false spots (flag of <5,000) and signal-to-noise values of <3 (as determined by the software) were excluded. Fold changes between *Id2*^−/−^ and wild-type samples were calculated for each of the resulting probe sets.

### Accession number(s).

Microarray data have been deposited in the Gene Expression Omnibus database under the accession code GSE43014.

### Statistical analysis.

Statistical analysis was performed using two-tailed Student's *t* tests to calculate *P* values. Error bars show standard errors of the means (SEM).

## Supplementary Material

Supplemental material

## References

[1] Zorn AM, Wells JM 2009 Vertebrate endoderm development and organ formation. Annu Rev Cell Dev Biol 25:221–251. doi:10.1146/annurev.cellbio.042308.113344. 19575677 PMC2861293

[2] Slack JM 2007 Metaplasia and transdifferentiation: from pure biology to the clinic. Nat Rev Mol Cell Biol 8:369–378. doi:10.1038/nrm2146. 17377526

[3] Yuasa Y 2003 Control of gut differentiation and intestinal-type gastric carcinogenesis. Nat Rev Cancer 3:592–600. doi:10.1038/nrc1141. 12894247

[4] Massari ME, Murre C 2000 Helix-loop-helix proteins: regulators of transcription in eucaryotic organisms. Mol Cell Biol 20:429–440. doi:10.1128/MCB.20.2.429-440.2000. 10611221 PMC85097

[5] Lasorella A, Benezra R, Iavarone A 2014 The ID proteins: master regulator of cancer stem cells and tumor aggressiveness. Nat Rev Cancer 14:77–91. doi:10.1038/nrc3638. 24442143

[6] Yang HY, Liu HL, Liu GY, Zhu H, Meng QW, Qu LD, Liu LX, Jiang HC 2011 Expression and prognostic values of Id-1 and Id-3 in gastric adenocarcinoma. J Surg Res 167:258–266. doi:10.1016/j.jss.2009.08.006. 20080245

[7] Wilson JW, Deed RW, Inoue T, Balzi M, Becciolini A, Faraoni P, Potten CS, Norton JD 2001 Expression of Id helix-loop-helix proteins in colorectal adenocarcinoma correlates with p53 expression and mitotic index. Cancer Res 61:8803–8810. 11751402

[8] Wice BM, Gordon JI 1998 Forced expression of Id-1 in the adult mouse small intestinal epithelium is associated with development of adenomas. J Biol Chem 273:25310–25319. doi:10.1074/jbc.273.39.25310. 9737997

[9] Russell RG, Lasorella A, Dettin LE, Iavarone A 2004 Id2 drives differentiation and suppresses tumor formation in the intestinal epithelium. Cancer Res 64:7220–7225. doi:10.1158/0008-5472.CAN-04-2095. 15492237

[10] Que J, Okubo T, Goldenring JR, Nam KT, Kurotani R, Morrisey EE, Taranova O, Pevny LH, Hogan BL 2007 Multiple dose-dependent roles for Sox2 in the patterning and differentiation of anterior foregut endoderm. Development 134:2521–2531. doi:10.1242/dev.003855. 17522155 PMC3625644

[11] Cheng H, Leblond CP 1974 Origin, differentiation and renewal of the four main epithelial cell types in the mouse small intestine. V. Unitarian theory of the origin of the four epithelial cell types. Am J Anat 141:537–561. 4440635 10.1002/aja.1001410407

[12] Su LK, Kinzler KW, Vogelstein B, Preisinger AC, Moser AR, Luongo C, Gould KA, Dove WF 1992 Multiple intestinal neoplasia caused by a mutation in the murine homolog of the APC gene. Science 256:668–670. doi:10.1126/science.1350108. 1350108

[13] Taketo MM, Edelmann W 2009 Mouse models of colon cancer. Gastroenterology 136:780–798. doi:10.1053/j.gastro.2008.12.049. 19263594

[14] Karam SM, Leblond CP 1993 Dynamics of epithelial cells in the corpus of the mouse stomach. I. Identification of proliferative cell types and pinpointing of the stem cell. Anat Rec 236:259–279. 8338232 10.1002/ar.1092360202

[15] James R, Erler T, Kazenwadel J 1994 Structure of the murine homeobox gene cdx-2. Expression in embryonic and adult intestinal epithelium. J Biol Chem 269:15229–15237. 7910823

[16] Silberg DG, Swain GP, Suh ER, Traber PG 2000 Cdx1 and cdx2 expression during intestinal development. Gastroenterology 119:961–971. doi:10.1053/gast.2000.18142. 11040183

[17] Fukamachi H, Mizuno T, Takayama S 1979 Epithelial-mesenchymal interactions in differentiation of stomach epithelium in fetal mice. Anat Embryol 157:151–160. doi:10.1007/BF00305155. 517763

[18] Beck F, Chawengsaksophak K, Waring P, Playford RJ, Furness JB 1999 Reprogramming of intestinal differentiation and intercalary regeneration in Cdx2 mutant mice. Proc Natl Acad Sci U S A 96:7318–7323. doi:10.1073/pnas.96.13.7318. 10377412 PMC22083

[19] Gao N, White P, Kaestner KH 2009 Establishment of intestinal identity and epithelial-mesenchymal signaling by Cdx2. Dev Cell 16:588–599. doi:10.1016/j.devcel.2009.02.010. 19386267 PMC2673200

[20] Grainger S, Savory JG, Lohnes D 2010 Cdx2 regulates patterning of the intestinal epithelium. Dev Biol 339:155–165. doi:10.1016/j.ydbio.2009.12.025. 20043902

[21] Raghoebir L, Bakker ER, Mills JC, Swagemakers S, Kempen MB, Munck AB, Driegen S, Meijer D, Grosveld F, Tibboel D, Smits R, Rottier RJ 2012 SOX2 redirects the developmental fate of the intestinal epithelium toward a premature gastric phenotype. J Mol Cell Biol 4:377–385. doi:10.1093/jmcb/mjs030. 22679103 PMC3523556

[22] Gontan C, de Munck A, Vermeij M, Grosveld F, Tibboel D, Rottier R 2008 Sox2 is important for two crucial processes in lung development: branching morphogenesis and epithelial cell differentiation. Dev Biol 317:296–309. doi:10.1016/j.ydbio.2008.02.035. 18374910

[23] Kuzmichev AN, Kim SK, D'Alessio AC, Chenoweth JG, Wittko IM, Campanati L, McKay RD 2012 Sox2 acts through Sox21 to regulate transcription in pluripotent and differentiated cells. Curr Biol 22:1705–1710. doi:10.1016/j.cub.2012.07.013. 22902753

[24] Kim BM, Buchner G, Miletich I, Sharpe PT, Shivdasani RA 2005 The stomach mesenchymal transcription factor Barx1 specifies gastric epithelial identity through inhibition of transient Wnt signaling. Dev Cell 8:611–622. doi:10.1016/j.devcel.2005.01.015. 15809042

[25] Kim BM, Miletich I, Mao J, McMahon AP, Sharpe PA, Shivdasani RA 2007 Independent functions and mechanisms for homeobox gene Barx1 in patterning mouse stomach and spleen. Development 134:3603–3613. doi:10.1242/dev.009308. 17855428

[26] Jayewickreme CD, Shivdasani RA 2015 Control of stomach smooth muscle development and intestinal rotation by transcription factor BARX1. Dev Biol 405:21–32. doi:10.1016/j.ydbio.2015.05.024. 26057579 PMC4529797

[27] Jen Y, Manova K, Benezra R 1996 Expression patterns of Id1, Id2, and Id3 are highly related but distinct from that of Id4 during mouse embryogenesis. Dev Dyn 207:235–252. doi:10.1002/(SICI)1097-0177(199611)207:3<235::AID-AJA1>3.0.CO;2-I. 8922523

[28] Batts LE, Polk DB, Debois RN, Kulessa. 2006 Bmp signaling is required for intestinal growth and morphogenesis. Dev Dyn 235:1563–1570. doi:10.1002/dvdy.20741. 16538672

[29] Rodriguez P, Da Silva S, Oxburgh L, Wang F, Hogan BL, Que J 2010 BMP signaling in the development of the mouse esophagus and forestomach. Development 137:4171–4176. doi:10.1242/dev.056077. 21068065 PMC2990208

[30] Miyazono K, Maeda S, Imamura T 2005 BMP receptor signaling: transcriptional targets regulation of signals, and signaling cross-talk. Cytokine Growth Factor Rev 16:251–263. doi:10.1016/j.cytogfr.2005.01.009. 15871923

[31] Nakahiro T, Kurooka H, Mori K, Sano K, Yokota Y 2010 Identification of BMP-responsive elements in the mouse Id2 gene. Biochem Biophys Res Commun 399:416–421. doi:10.1016/j.bbrc.2010.07.090. 20674548

[32] Danesh SM, Villasenor A, Chong D, Soukup C, Cleaver O 2009 BMP and BMP receptor expression during murine organogenesis. Gene Expr Patterns 9:255–265. doi:10.1016/j.gep.2009.04.002. 19393343 PMC2709213

[33] Sherwood RI, Maehr R, Mazzoni EO, Melton DA 2011 Wnt signaling specifies and patterns intestinal endoderm. Mech Dev 128:387–400. doi:10.1016/j.mod.2011.07.005. 21854845 PMC3223331

[34] Kim BM, Mao J, Taketo MM, Shivdasani RA 2007 Phases of canonical Wnt signaling during the development of mouse intestinal epithelium. Gastroenterology 133:529–538. doi:10.1053/j.gastro.2007.04.072. 17681174

[35] DasGupta R, Fuchs E 1999 Multiple roles for activated LEF/TCF transcription complexes during hair follicle development and differentiation. Development 126:4557–4568. 10498690 10.1242/dev.126.20.4557

[36] Gómez-Skarmeta JL, Modolell J 2002 Iroquois genes: genomic organization and function in vertebrate neural development. Curr Opin Genet Dev 12:403–408. doi:10.1016/S0959-437X(02)00317-9. 12100884

[37] Houweling AC, Dildrop R, Peters T, Mummenhoff J, Moorman AF, Rüther U, Christoffels VM 2001 Gene and cluster-specific expression of the Iroquois family members during mouse development. Mech Dev 107:169–174. doi:10.1016/S0925-4773(01)00451-8. 11520674

[38] Sherwood RI, Chen TY, Melton DA 2009 Transcriptional dynamics of endodermal organ formation. Dev Dyn 238:29–42. doi:10.1002/dvdy.21810. 19097184 PMC3756511

[39] Madison BB, Dunbar L, Qiao XT, Braunstein K, Braunstein E, Gumucio DL 2002 Cis elements of the villin gene control expression in restricted domains of the vertical (crypt) and horizontal (duodenum, cecum) axes of the intestine. J Biol Chem 277:33275–33283. doi:10.1074/jbc.M204935200. 12065599

[40] Yokota Y, Mansouri A, Mori S, Sugawara S, Adachi S, Nishikawa S, Gruss P 1999 Development of peripheral lymphoid organs and natural killer cells depends on the helix-loop-helix inhibitor Id2. Nature 397:702–706. doi:10.1038/17812. 10067894

[41] Mori S, Nishikawa SI, Yokota Y 2000 Lactation defect in mice lacking the helix-loop-helix inhibitor Id2. EMBO j 19:5772–5781. doi:10.1093/emboj/19.21.5772. 11060028 PMC305805

[42] Sugai M, Gonda H, Kusunoki T, Katakai T, Yokota Y, Shimizu A 2003 Essential role of Id2 in negative regulation of IgE class switching. Nat Immunol 4:25–30. doi:10.1038/ni874. 12483209

[43] Biyajima K, Kakizaki F, Shen X, Mori K, Sugai M, Taketo MM, Yokota Y 2015 Id2 deletion attenuates Apc-deficient ileal tumor formation. Biol Open 4:993–1001. doi:10.1242/bio.012252. 26163528 PMC4542283

[44] Girardi M 2006 Immunosurveillance and immunoregulation by gammadelta T cells. J Investig Dermatol 126:25–31. doi:10.1038/sj.jid.5700003. 16417214

[45] Kim JK, Takeuchi M, Yokota Y 2004 Impairment of intestinal intraepithelial lymphocytes in Id2 deficient mice. Gut 53:480–486. doi:10.1136/gut.2003.022293. 15016739 PMC1774007

[46] Lyden D, Young AZ, Zagzag D, Yan W, Gerald W, O'Reilly R, Bader BL, Hynes RO, Zhuang Y, Manova K, Benezra R 1999 Id1 and Id3 are required for neurogenesis, angiogenesis and vascularization of tumour xenografts. Nature 401:670–677. doi:10.1038/44334. 10537105

[47] Li HJ, Ray SK, Singh NK, Johnston B, Leiter AB 2011 Basic helix-loop-helix transcription factors and enteroendocrine cell differentiation. Diabetes Obes Metab 13(Suppl 1):5–12. doi:10.1111/j.1463-1326.2011.01438.x. 21824251 PMC3467197

[48] Jenny M, Uhl C, Roche C, Duluc I, Guillermin V, Guillemot F, Jensen J, Kedinger M, Gradwohl G 2002 Neurogenin3 is differentially required for endocrine cell fate specification in the intestinal and gastric epithelium. EMBO J 21:6338–6347. doi:10.1093/emboj/cdf649. 12456641 PMC136953

[49] Lee CS, Perreault N, Brestelli JE, Kaestner KH 2002 Neurogenin 3 is essential for the proper specification of gastric enteroendocrine cells and the maintenance of gastric epithelial cell identity. Genes Dev 16:1488–1497. doi:10.1101/gad.985002. 12080087 PMC186338

[50] López-Díaz L, Jain RN, Keeley TM, VanDussen KL, Brunkan CS, Gumucio DL, Samuelson LC 2007 Intestinal Neurogenin 3 directs differentiation of a bipotential secretory progenitor to endocrine cell rather than goblet cell fate. Dev Biol 309:298–305. doi:10.1016/j.ydbio.2007.07.015. 17706959 PMC2679162

[51] van Tuyl M, Liu J, Groenman F, Ridsdale R, Han RN, Venkatesh V, Tibboel D, Post M 2006 Iroquois genes influence proximo-distal morphogenesis during rat lung development. Am J Physiol Lung Cell Mol Physiol 290:L777–L789. doi:10.1152/ajplung.00293.2005. 16299054

[52] He W, Jia Y, Takimoto K 2009 Interaction between transcription factors Iroquois proteins 4 and 5 controls cardiac potassium channel Kv4.2 gene transcription. Cardiovasc Res 81:64–71. doi:10.1093/cvr/cvn259. 18815185 PMC2721642

[53] Gaborit N, Sakuma R, Wylie JN, Kim KH, Zhang SS, Hui CC, Bruneau BG 2012 Cooperative and antagonistic roles for Irx3 and Irx5 in cardiac morphogenesis and postnatal physiology. Development 139:4007–4019. doi:10.1242/dev.081703. 22992950 PMC3472592

[54] Li D, Sakuma R, Vakili NA, Mo R, Puviindran V, Deimling S, Zhang X, Hopyan S, Hui CC 2014 Formation of proximal and anterior limb skeleton requires early function of Irx3 and Irx5 and is negatively regulated by Shh signaling. Dev Cell 29:233–240. doi:10.1016/j.devcel.2014.03.001. 24726282

[55] Smemo S, Tena JJ, Kim KH, Gamazon ER, Sakabe NJ, Gomez-Marin C, Aneas I, Credidio FL, Sobreira DR, Wasserman NF, Lee JH, Puviindran V, Tam D, Shen M, Son JE, Vakili NA, Sung HK, Naranjo S, Acemel RD, Manzanares M, Nagy A, Cox NJ, Hui CC, Gomez-Skarmeta JL, Nobrega MA 2014 Obesity-associated variants within FTO form long-range functional connections with IRX3. Nature 507:371–375. doi:10.1038/nature13138. 24646999 PMC4113484

[56] Ragvin A, Moro E, Fredman D, Navratilova P, Drivenes O, Engstrom PG, Alonso ME, de la Calle Mustienes E, Gomez Skarmeta JL, Tavares MJ, Casares F, Manzanares M, van Heyningen V, Molven A, Njolstad PR, Argenton F, Lenhard B, Becker TS 2010 Long-range gene regulation links genomic type 2 diabetes and obesity risk regions to HHEX, SOX4, and IRX3. Proc Natl Acad Sci U S A 107:775–780. doi:10.1073/pnas.0911591107. 20080751 PMC2818943

[57] Claussnitzer M, Dankel SN, Kim KH, Quon G, Meuleman W, Haugen C, Glunk V, Sousa IS, Beaudry JL, Puviindran V, Abdennur NA, Liu J, Svensson PA, Hsu YH, Drucker DJ, Mellgren G, Hui CC, Hauner H, Kellis M 2015 FTO obesity variant circuitry and adipocyte browning in humans. N Engl J Med 373:895–907. doi:10.1056/NEJMoa1502214. 26287746 PMC4959911

[58] Bonnard C, Strobl AC, Shboul M, Lee H, Merriman B, Nelson SF, Ababneh OH, Uz E, Guran T, Kayserili H, Hamamy H, Reversade B 2012 Mutations in IRX5 impair craniofacial development and germ cell migration via SDF1. Nat Genet 44:709–713. doi:10.1038/ng.2259. 22581230

[59] Mowry RW 1963 The special value of methods that color both acidic and vicinal hydroxyl groups in the histochemical study of mucins with revised directions for the colloidal iron stain, the use of alcian blue 8GX and their combinations with the periodic acid-Schiff reaction. Ann N Y Acad Sci 106:402–423. doi:10.1111/j.1749-6632.1963.tb16654.x.

[60] Falk P, Roth KA, Gordon JI 1994 Lectins are sensitive tools for defining the differentiation programs of mouse gut epithelial cell lineages. Am J Physiol 266:G987–G1003. 8023947 10.1152/ajpgi.1994.266.6.G987

[61] Morita S, Kojima T, Kitamura T 2000 Plat-E: an efficient and stable system for transient packaging of retroviruses. Gene Ther 7:1063–1066. doi:10.1038/sj.gt.3301206. 10871756

[62] Watanabe Y, Inoue K, Okuyama-Yamamoto A, Nakai N, Nakatani J, Nibu K, Sato N, Iiboshi Y, Yusa K, Kondoh G, Takeda J, Terashima T, Takumi T 2009 Fezf1 is required for penetration of the basal lamina by olfactory axons to promote olfactory development. J Comp Neurol 515:565–584. doi:10.1002/cne.22074. 19479999

