# Supplementary material for: Id2 Determines Intestinal Identity through Repression of the Foregut Transcription Factor Irx5
Source: Mol Cell Biol. 2018 Apr 16;38(9):e00250-17. doi: 10.1128/MCB.00250-17 (PMC5902590; doi:10.1128/MCB.00250-17)
Supplement: Supplemental material [file supp_38_9_e00250-17__index.html]

Supplemental material 

# Id2 Determines Intestinal Identity through Repression of the Foregut Transcription Factor Irx5

## Supplemental material

- Supplemental file 1 -

  Supplemental text; Fig. S1 and S2 (Histopathology [S1] and characterization [S2] of intestinal lesions of *Id2*−/− mice), S3 (Immunohistochemistry of squamous epithelia in small intestine of *Id2*−/− mice), S4 (Gastric cells in intestinal tumor of *Id2*−/− mice), S5 (*Id2*−/− midgut mesenchyme induction of gastric epithelial cells), S6 (BMP-Smad signaling in *Id2*−/− embryonic midgut), S7 (Ectopic *Id2* expression induction of intestinal epithelial cells), and S8 (Irx5 expression in *Irx5*-Tg mice); and Tables S1 (Development of gastric tumors in *Id2*−/− mice), S2 (Histopathological classification), S3 (Down- and upregulated genes in *Id2*−/− intestine), S4 and S5 (Tumor development in *Irx5*-Tg mice), S6 (Antibodies for immunostaining), and S7 (PCR primers)

  PDF, 3.2M
