# Supplementary material for: Id2 Determines Intestinal Identity through Repression of the Foregut Transcription Factor Irx5
Source: Mol Cell Biol. 2018 Apr 16;38(9):e00250-17. doi: 10.1128/MCB.00250-17 (PMC5902590; doi:10.1128/MCB.00250-17)
Supplement: Supplemental material [file MCB.00250-17_zmb999101743s1.pdf]

Supplemental Information

Materials and Methods

Co-culture of midgut endoderm and mesenchyme

Endodermal cells and mesenchymal cells were prepared as described by Shimizu with minor modifications (*63*). The distal two-thirds of E13.5 midgut organs were excised in ice-cold HBSS and treated with 0.1% (w/v) collagenase A (Roche) containing Keratinocyte Basal Medium 2 (KBM2) and 6 nM $CaCl_2$ (KGM2) (Promo Cell GmbH, Heidelberg, Germany) for 60 min at 37°C. Endodermal tubes and mesenchyme were separated by gentle pipetting. The collected endodermal cells from 1 gut and mesenchyme from 1 gut were suspended by pipetting to cause fragmentation in KGM2 medium and mixed in a 1.5-mL tube. Mixed endodermal cells and mesenchymal cells were seeded into type I-collagen-coated 48-well plates (IWAKI Glass, Tokyo, Japan), and cultured at 5% CO_2 at 37°C for 7 days in KGM2 medium.

Western blotting

E13.5 midgut tissues were homogenized in RIPA buffer (50 mM Tris-HCl, 150 mM NaCl, 1% Triton X-100, 0.5% sodium deoxycholate, 1 mM EDTA, 0.1% SDS) supplemented with Proteinase Inhibitor cocktail

and Phosphatase Inhibitor cocktail (Nacalai Tesque, Osaka, Japan). Lysate were separated by 11–15% SDS-PAGE and transferred to 0.2-µm nitrocellulose membranes (GE Healthcare, Little Chalfont, UK). After blocking with 3% skim milk-TBST or 2% BSA-TBST (phospho. Smad1/5/8), membranes were incubated with primary antibodies at 4°C overnight. After washing with TBST, the membranes were incubated with horseradish peroxidase-conjugated secondary antibodies for 1 h at room temperature. Immunoreactive bands were visualized by the chemiluminescence method using Amersham ECL Prime Western Blotting Detection Reagent (GE Healthcare). Antibodies used for western blotting analysis are listed in Supplemental Table S6.

Immunohistochemistry

For immunohistochemical analysis, tissues were fixed with 4% paraformaldehyde and paraffin-embedded using standard methods. Next, 4-µm-thick serial paraffin sections were stained with antibodies according to standard methods. Immunofluorescent staining of cultured cells was performed as described previously (*64*). Antisera against mouse Id2 peptide (AA 109 - 123) were raised by immunization of rabbits. The immunoglobulin G fraction of antisera was subject to affinity purification against the recombinant glutathione S-transferase (GST) fusion full-length Id2 protein produced by *E. coli.* coupled to a column

(HiTrap NHS-activated, Amersham Pharmacia Biotech, Orsay, France). Antibodies used for immunohistochemical analysis are listed in Supplemental Table S6.

Reference

63. **Shimizu M, Minakuchi K, Tsuda A, Hiroi T, Tanaka N, Koga J, Kiyono H.** 2001. Role of stem cell factor and c-kit signaling in regulation of fetal intestinal epithelial cell adhesion to fibronectin. Exp Cell Res **266:**311-22.

64. **Mori K, Furusawa T, Okubo T, Inoue T, Ikawa S, Yanai N, Mori KJ, Obinata M.** 2003. Genome structure and differential expression of two isoforms of a novel PDZ-containing myosin (MysPDZ) (Myo18A). J Biochem **133:**405-413.

Supplementary Figure Legends

Fig. S1. Histopathology of the intestinal lesions of *Id2*$^{-/-}$ mice. Hematoxylin and eosin (HE) staining of representative intestinal lesions is shown based on a histopathological diagnosis. (Upper left) simple heterotopic lesion; (Upper right) hyperplastic epithelium; (Middle upper and middle) Adenoma, low grade, with different cellular atypia; (Middle lower) adenoma, high grade, with severe cellular dysplasia; (Lower) adenocarcinoma with mucosal infiltration. Higher magnification of boxed area in adenoma and adenocarcinoma are shown in right panels. HE staining of a representative hyperplastic epithelium is shown in Fig. 1C. Scale bars: 100 μm.

Figure S2. Characterization of the intestinal lesions of *Id2*$^{-/-}$ mice. (**A**) Frequency and site of lesions along the rostral to caudal axis of the small intestine. The small intestine was subdivided into 20 parts along the rostral to caudal axis, and the sites of 176 lesions (from 43 mice) were counted. (**B**) Correlation between size and histopathology of lesions (n=51) in *Id2*$^{-/-}$ mice (aged 30–40 weeks).

Fig. S3. Immunohistochemistry of squamous epithelia in the small intestine of *Id2*$^{-/-}$ mice. High-power views of the boxed regions are shown in the respective right panels. (Upper) Immunohistochemistry for p63. Heterotopic squamous epithelia stained positive for p63. (Lower) Immunohistochemistry for cytokeratin (CK) 14. Squamous epithelia developed in the small intestine also stained positive for CK14. (Upper) and (Lower) are serial sections. Scale bars; 200 μm.

Fig. S4. Gastric cell in the intestinal tumor of *Id2*$^{-/-}$ mice. (**A**) Immunohistochemistry using an anti-TFF2 antibody to detect mucus neck cells in a tumor. Right panels show normal stomach (corpus) epithelium. Mucus neck cells are located in the neck region, and contained secretory granules. (**B**) Immunohistochemistry for Muc5AC. The apical surface of tumor cells stained positive for Muc5AC. (**C**) Immunohistochemistry for Cdx2. Tumor cells are deficient in Cdx2 expression. Right panels show the normal jejunum. (**B**) and (**C**) are serial sections. Scale bars: 200 μm.

Fig. S5 *Id2*$^{-/-}$ midgut mesenchyme induces gastric epithelial cells. (**A**) Preparation of fetal midgut endoderm and mesenchyme. The midgut tissue fragment isolated from the embryo was incubated in 0.1% collagenase A containing medium at 37°C for 1 h. Endoderm and mesenchyme were separated by gentle pipetting. (**B**) RT-PCR analysis of the isolated cells. End., Endoderm. Mes., Mesenchyme. (**C**) Co-culture image of endoderm cells and mesenchymal cells. (Upper) Bright-field image. (Lower) Immunostaining for Cdh1. Arrowhead indicates endodermal cell colony. (**D**) RT-PCR analysis of co-cultured cells. Upper panel indicates the genotype of co-cultured endoderm and mesenchyme. +/+; *Id2*$^{+/+}$, −/−; *Id2*$^{-/-}$, (−); empty of endoderm. (**E**) Quantitative analysis of Cdx2 expression in co-cultured endodermal cells (n = 4). Data were normalized relative to *Cdh1* amplification. $^{**}P < 0.01$.

Fig. S6. BMP-Smad signaling in *Id2*$^{-/-}$ embryonic midgut. (**A**) qRT-PCR analysis of Bmp signaling components. n.s.; not significant (**B**) Western blotting for Bmp-Smad signaling mediator and its transcriptional target molecule. P-Smad1/5/8; Phosphorylated Smad1/5/8. Actin served as an internal control. (**C**) (Left) E12.5 *Id2*$^{-/-}$ small intestines

were stained for Cdx2 and pSmad1/5/8. (Right) High-magnification image of boxed region shown in left panel. Scale bar, 50 µm (**D**) qRT-PCR analysis of Bmp-Smad target genes (n = 6).

Fig. S7, Ectopic *Id2* expression induced intestinal epithelial cells in the developing *Id2*$^{-/-}$ stomach. (**A**) Immunofluorescence for Id2 to recovered *Id2*$^{-/-}$ stomach. Upper panel shows transduced genes. *gfp*, stomach transduced with EGFP cDNA; *Id2*, stomach transduced with *Id2* cDNA. Scale bar, 50 µm (**B**) Immunohistochemistry of recovered stomachs. upper and lower panels are serial sections. Transduced genes are indicated on top. Induced intestinal epithelial cells were positive for Cdx2 (brown). AB stained goblet cells blue. Scale bar, 50 µm (**C**) qRT-PCR analysis of recovered Cdx2- and AB-positive stomach tissue (Id2-1, -2, -3). graft, engrafted tissues. (**D**) RT-PCR analysis of recovered stomachs. St, wild-type stomach at P7, S.Int, wild-type small intestine at P7; *Actb* served as an internal control.

Fig. S8. Irx5 expression in *Irx5*-Tg mice. (**A**) *Irx5* expression measured by quantitative RT-PCR in E13.5 *Irx5*-Tg small intestine. Genotypes of wild-type (+/+) and *Irx5*-Tg (Tg/+) embryonic intestines are shown as black and gray, respectively. n=6. $^{**}P < 0.01$. (**B**) Immunohistochemistry of the small intestine using an anti-Irx5 antibody. Irx5 expression was detected in the intestinal epithelial cells of *Irx5*-Tg (Tg/+) mice, but not in those of wild-type (+/+) mice at E15.5. Scale bars; 50 µm.

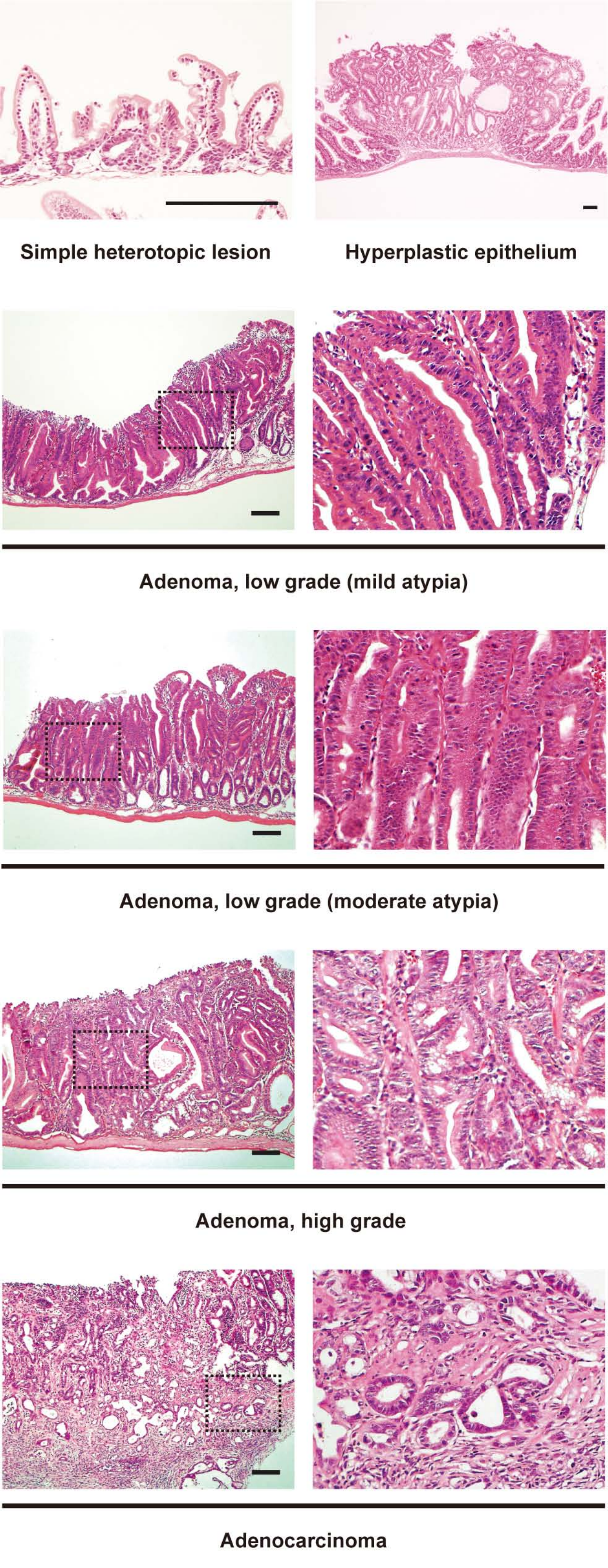
Simple heterotopic lesion
Hyperplastic epithelium
Adenoma, low grade (mild atypia)
Adenoma, low grade (moderate atypia)
Adenoma, high grade
Adenocarcinoma

Fig. S1

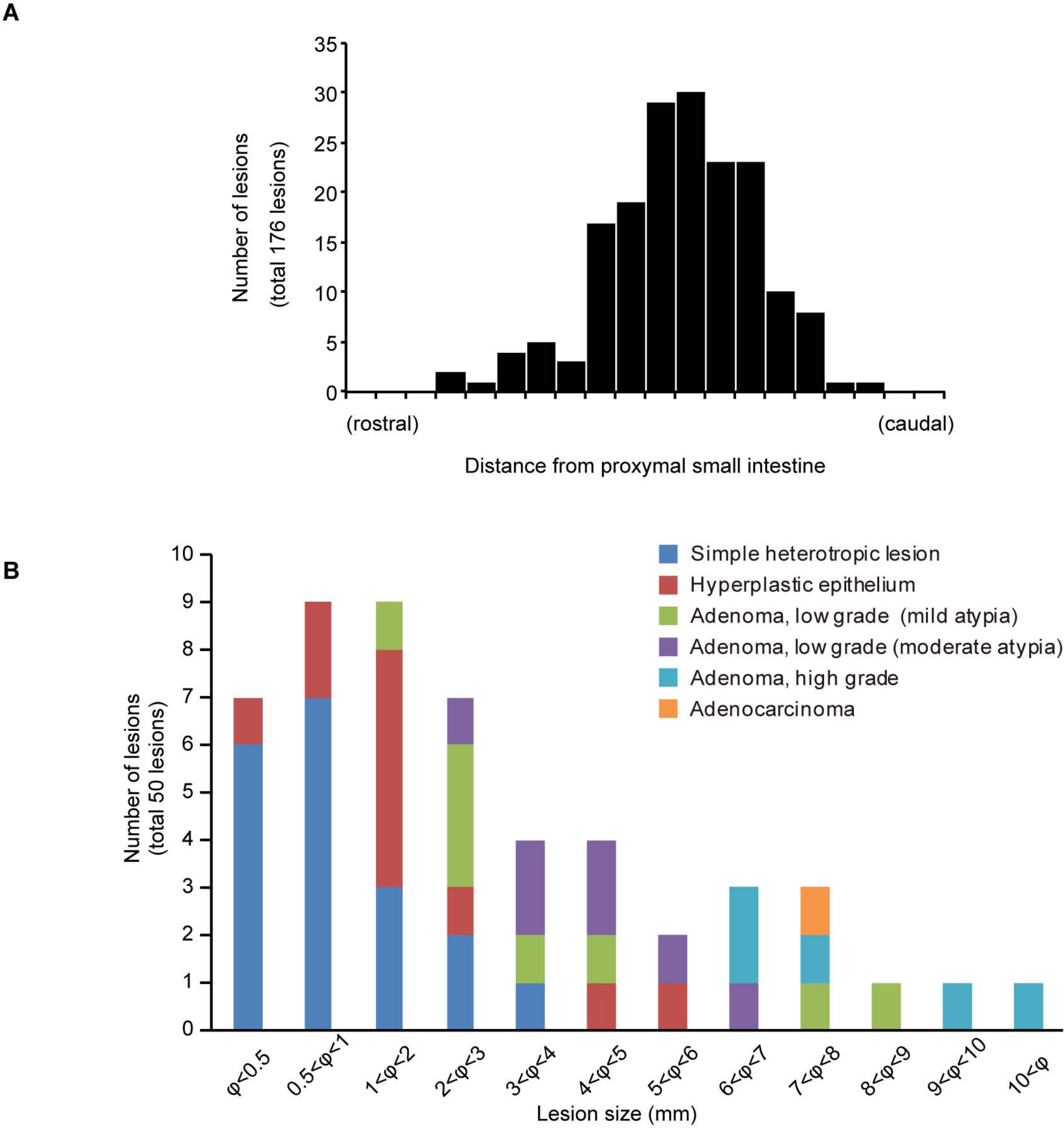
A
Number of lesions
(total 176 lesions)
35
30
25
20
15
10
5
0
(rostral)
(caudal)
Distance from proxymal small intestine
B
Number of lesions
(total 50 lesions)
10
9
8
7
6
5
4
3
2
1
0
Simple heterotropic lesion
Hyperplastic epithelium
Adenoma, low grade (mild atypia)
Adenoma, low grade (moderate atypia)
Adenoma, high grade
Adenocarcinoma
φ<0.5
0.5<φ<1
1<φ<2
2<φ<3
3<φ<4
4<φ<5
5<φ<6
6<φ<7
7<φ<8
8<φ<9
9<φ<10
10<φ
Lesion size (mm)

Fig. S2

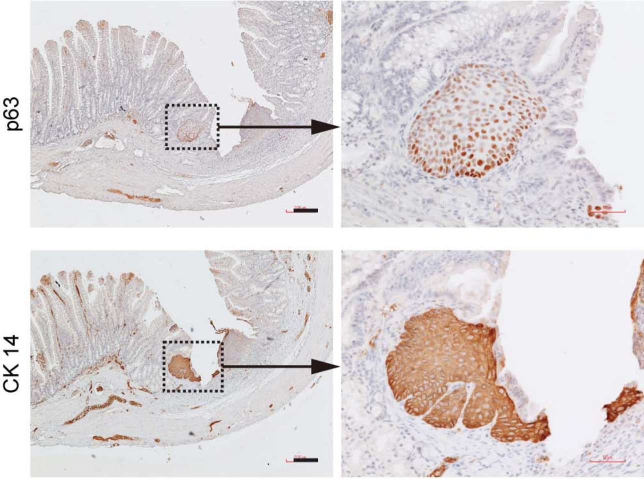

Fig. S3

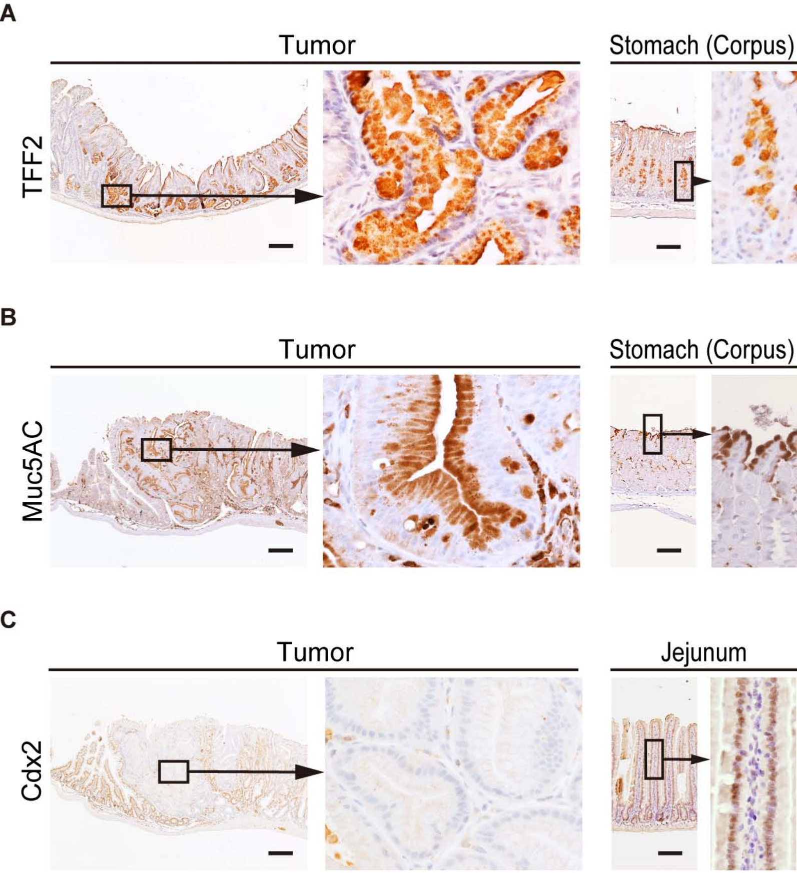
A
Tumor
Stomach (Corpus)
TFF2
B
Tumor
Stomach (Corpus)
Muc5AC
C
Tumor
Jejunum
Cdx2

Fig. S4

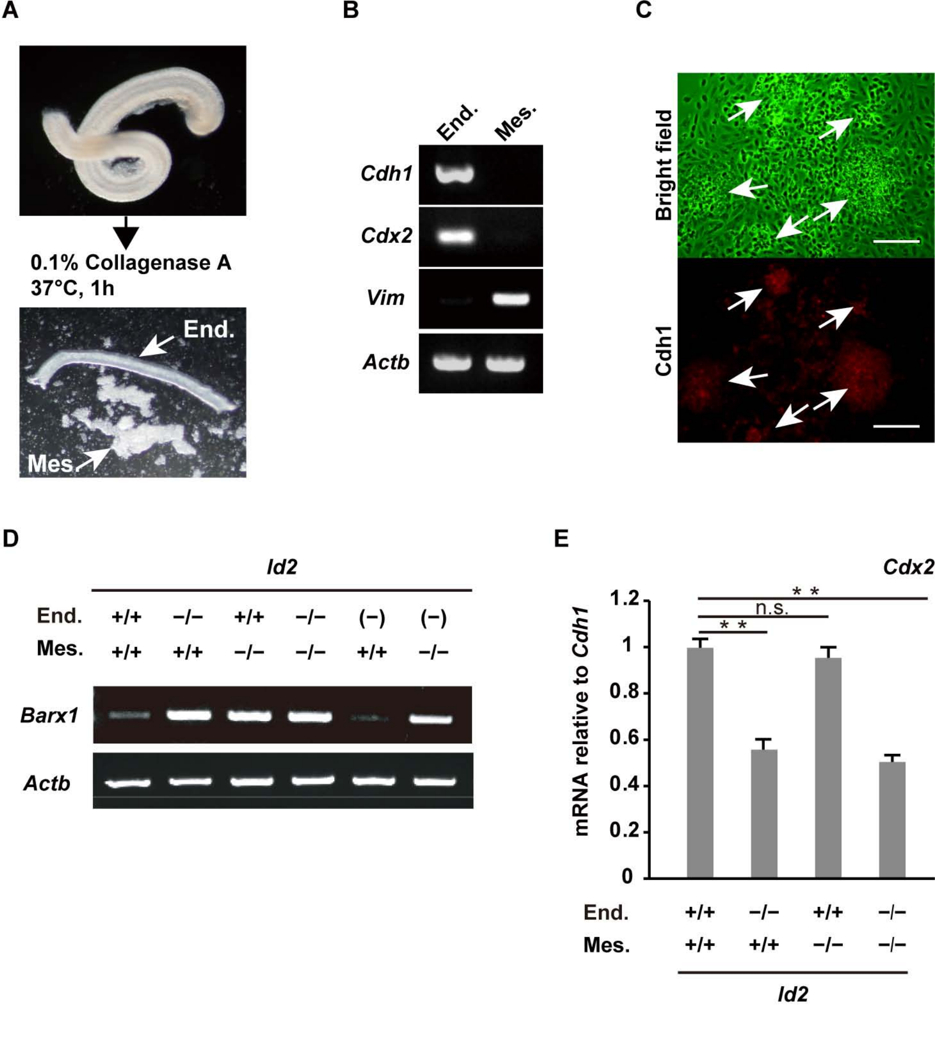
A
0.1% Collagenase A
37°C, 1h
End.
Mes.
B
End.
Mes.
Cdh1
Cdx2
Vim
Actb
C
Bright field
Cdh1
D
Id2
End. +/+ −/− +/+ −/− (−) (−)
Mes. +/+ +/+ −/− −/− +/+ −/−
Barx1
Actb
E
Cdx2
mRNA relative to Cdh1
1.2
1
0.8
0.6
0.4
0.2
0
n.s.
* *
* *
End. +/+ −/− +/+ −/−
Mes. +/+ +/+ −/− −/−
Id2

Fig. S5

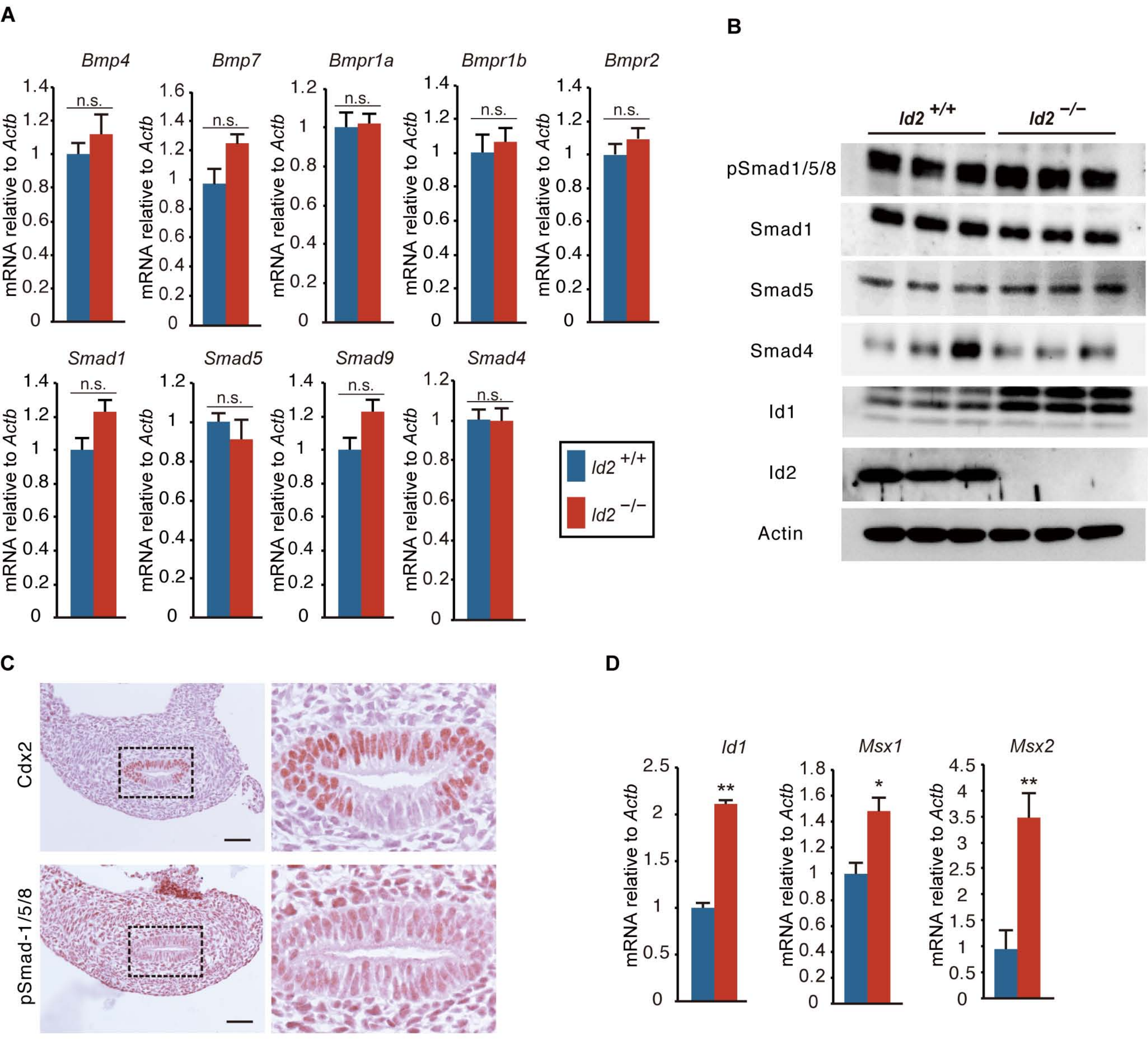

A
Bmp4
Bmp7
Bmpr1a
Bmpr1b
Bmpr2
Smad1
Smad5
Smad9
Smad4
n.s.
mRNA relative to Actb
Id2 +/+
Id2 −/−
B
pSmad1/5/8
Smad1
Smad5
Smad4
Id1
Id2
Actin
C
Cdx2
pSmad-1/5/8
D
Id1
Msx1
Msx2
**
*

Fig. S6

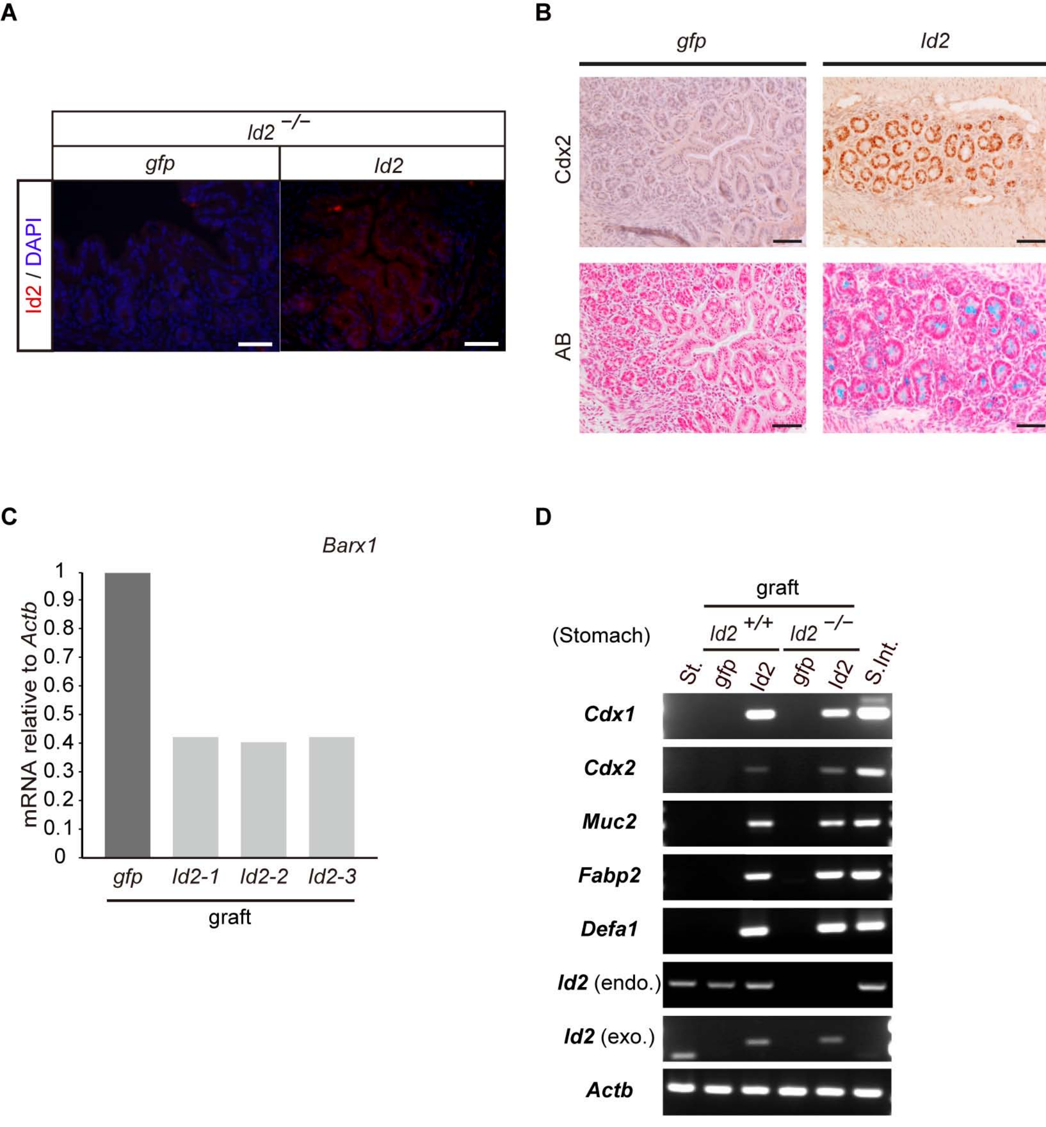
A
Id2 −/−
gfp
Id2
Id2 / DAPI
B
gfp
Id2
Cdx2
AB
C
Barx1
mRNA relative to Actb
1
0.9
0.8
0.7
0.6
0.5
0.4
0.3
0.2
0.1
0
gfp
Id2-1
Id2-2
Id2-3
graft
D
graft
(Stomach)
Id2 +/+
Id2 −/−
St.
gfp
Id2
gfp
Id2
S.Int.
Cdx1
Cdx2
Muc2
Fabp2
Defa1
Id2 (endo.)
Id2 (exo.)
Actb

Fig. S7

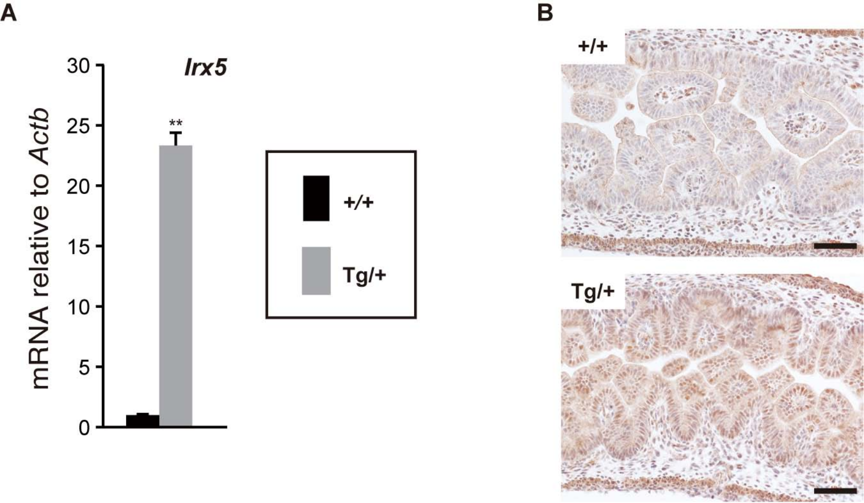

Fig. S8

Table S1

Genotype	*Id2*$^{-/-}$	*Id2*$^{+/-}$	*Id2*$^{+/+}$
Tumor incidence	78/81	0/17	0/16
Tumors number (mean ± SD)	4.3 ± 2.9	0	0

Table S2

Histopathological classifycation	(n=51)
Simple heterotopic lesion	19/51
Hyperplastic epithelium	11/51
Adenoma, low grade (mild atypia)	8/51
Adenoma, low grade (moderate atypia)	7/51
Adenoma, high grade	5/51
Adenocarcinoma	1/51

Table S3

Genes down-regulated in E13.5 *Id2*$^{-/-}$ small intestine

(*Id2*$^{+/+}$ / *Id2*$^{-/-}$ > 2.0)

Gene Symbol	Gene Name	Fold change	GO Molecular Function
Cbln2	cerebellin 2 precursor protein	5.38	-
Sult1d1	sulfotransferase family 1D, member 1	5.19	tyrosine-ester sulfotransferase activity
Spink3	serine peptidase inhibitor, Kazal type 3	4.91	endopeptidase inhibitor activity
0610012H03Rik	RIKEN cDNA 0610012H03 gene	4.88	-
Anxa13	annexin A13	4.66	calcium ion binding
Kynu	kynureninase (L-kynurenine hydrolase)	4.41	hydrolase activity
Muc13	mucin 13, epithelial transmembrane	4.25	-
Ppp1r1b	protein phosphatase 1, regulatory (inhibitor) subunit 1B	3.88	protein phosphatase type 1 regulator activity
Lingo1	leucine rich repeat and Ig domain containing 1	3.74	-
Myl1	myosin, light polypeptide 1	3.42	motor activity
2510049J12Rik	RIKEN cDNA 2510049J12 gene	3.38	-
Bspry	B-box and SPRY domain containing	3.36	zinc ion binding
Fabp1	fatty acid binding protein 1, liver	3.15	fatty acid binding
Hapln2	hyaluronan and proteoglycan link protein 2	3.04	hyaluronic acid binding
BC030870	cDNA sequence BC030870	2.76	-
2610044O15Rik	RIKEN cDNA 2610044O15 gene	2.69	-
Ifi203	interferon activated gene 203	2.51	-
Slc27a2	solute carrier family 27 (fatty acid transporter), member 2	2.50	long-chain-fatty-acid-CoA ligase activity
Foxq1	forkhead box Q1	2.39	transcription factor activity
Faim3	Fas apoptotic inhibitory molecule 3	2.38	-

Genes up-regulated in E13.5 *Id2*$^{-/-}$ small intestine

(*Id2*$^{+/+}$ / *Id2*$^{-/-}$ < 0.5)

Gene Symbol	Gene Name	Fold change	GO Molecular Function
Irx5	Iroquois related homeobox 5 (Drosophila)	0.128	transcription factor activity
Adcy8	adenylate cyclase 8	0.155	metal ion binding
Cym	Chymosin	0.207	-
Foxa2	forkhead box A2	0.245	transcription factor activity
Ccdc96	coiled-coil domain containing 96	0.252	-
Krt1-15	keratin complex 1, acidic, gene 15	0.279	structural constituent of cytoskeleton
Irx3	Iroquois related homeobox 3 (Drosophila)	0.311	transcription factor activity
Olfr432	olfactory receptor 432	0.317	receptor activity
Traf6	Tnf receptor-associated factor 6	0.323	signal transducer activity
C030016D13Rik	RIKEN cDNA C030016D13 gene	0.328	-
Ocrl	oculocerebrorenal syndrome of Lowe	0.382	phosphoinositide 5-phosphatase activity
Wdr86	WD repeat domain 86	0.419	-
Cacng7	calcium channel, voltage-dependent, gamma subunit 7	0.431	calcium ion binding
Olfr1337	olfactory receptor 1337	0.433	receptor activity

Table S4

Genotype	+/+	Tg/+
Tumor incidence	0/23	11/68
Tumors number (mean ± SD)	0	1.2 ± 0.4

Table S5

Histopathological classifycation	(n=13)
Simple heterotopic lesion	2/13
Hyperplastic epithelium	6/13
Adenoma, low grade (mild atypia)	3/13
Adenoma, low grade (moderate atypia)	2/13
Adenoma, high grade	0/13
Adenocarcinoma	0/13

Table S6

Antigen	Antiserum	Souce	Clone	Dilution (IHC)	Dilution (WB)
β-Catenin	Mouse monoclonal anti β-Catenin	BD Transduction laboratories	14	1:100	
H^+/K^+ -ATPase α subunit	Mouse monoclonal H^+/K^+ -ATPase α subunit	MBL	1H9	1:250	
Pepsinogen C	Sheep polyclonal anti Pepsinogen II	Abcam (ab9013)		1:500	
Lysozyme	Rabbit polyclonal anti Lysozyme	DAKO (EC3.2.1.17)		1:200	
Cdx2	Mouse monoclonal anti Cdx2	BioGenex	CDX2-88	1:100	
Cytokeratin 14	Mouse monoclonal anti Keratin 14	Neomarkers	LL002	1:300	
Sox2	Rabbit polyclonal anti Sox2	Millipore (AB5603)		1:500	
p63	Mouse monoclonal anti p63	Santacruz (sc-8431)	A4A	1:1000	
Barx1	Rabbit polyclonal anti Barx1	Bioss (bs-8521R)		1:50	
GFP	Rabbit polyclonal anti GFP	MBL (598)		1:2000	
Irx5	Rabbit polyclonal anti Irx5	Santacruz (sc-98397)		1:100	
Cdh1	Mouse monoclonal anti Cdh1	TaKaRa (M107)	ECCD-2	1:100	
Phospho. Smad1/5/8	Rabbit polyclonal anti phospho Smad1/5/8 (Ser463/465)	Millipore (AB3848-I)		1:100	1:500
Smad1	Rabbit polyclonal anti Smad1	Zymed(38-5400)			1:250
Smad5	Rabbit polyclonal anti Smad5	CST (9517)			1:1000
Smad4	Mouse monoclonal anti Smad4	Santacruz(sc-7966)	B-8		1:200
Id1	Rabbit polyclonal anti Id1	Santacruz(sc-488)	C-20		1:200
Id2	Rabbit polyclonal anti Id2	Yokota et.al			1:2000
Actin	Rabbit polyclonal anti Actin	Sigma (A2066)			1:500

Table S7

Gene Symbol	Gene description	Genebank accession No.	Forward Primer (5' to 3')	Reverse Primer (5' to 3')	Size (bp)	Detection
Cdx1	caudal type homeobox 1	NM_009880	GGACGCCCTACGAATGGAT	CCCAGAAGGCCAGCATTAGT	376	Conventional RT-PCR
Cdx2	caudal type homeobox 2	NM_007673	CTGCTACGGCGAACTTGGAC	CAGCCGCTGATGGTCTGTGT	446	Conventional RT-PCR
Muc2	mucin 2	NM_023566	TGGCCCTGAAGAAGAACC	GGGCACACAGGAATAAACTG	451	Conventional RT-PCR
Fabp2	fatty acid binding protein 2, intestinal	NM_007980	GTAGACCGGAACGAGAACTATG	TAGCTTTGACAAGGCTGGAGAC	501	Conventional RT-PCR
Defa1	defensin, alpha 1	NM_010031	AGCAGCCAGGAGAAGAGGAC	GTCATCAGGCACCAGCATC	251	Conventional RT-PCR
Muc1	mucin 1, transmembrane	NM_013605	GGCTCAGCCACCAGTCCA	TGGGCAGAGGGAGGGAACT	719	Conventional RT-PCR
Muc5ac	mucin 5, subtypes A and C, tracheobronchial/gastric	NM_010844	GAGGGCCCAGTGAGCATCTCC	TGGGACAGCAGCAGTATTCAGT	361	Conventional RT-PCR
Atp4b	ATPase, H+/K+ exchanging, beta polypeptide	NM_009724	CATGCACGGCCACTTCCAT	CATGCCAGGGTGACCAGTGTTA	253	Conventional RT-PCR
Tff1	trefoil factor 1	NM_009362	GCACAAGGTGATCTGTGTCCTC	CCCGGACACTGTCATCAAAAC	194	Conventional RT-PCR
Tff2	trefoil factor 2	NM_009363	GTGAGCAGTGCTTTGATCTTGG	GGCACTTCAAAGATCAGGTTGG	202	Conventional RT-PCR
Gif	gastric intrinsic factor	NM_008118	GAACTGGTCACCTTCAAGC	AGCAGATCAACCCCTCTCA	630	Conventional RT-PCR
Pgc	pepsinogen C	NM_025973	TGACCCTGGCCAGAAATACC	GCCCATGATCCCGTCAAA	409	Conventional RT-PCR
Id2 (endogenous)	Inhibitor of DNA binding 2	NM_010496	AGCATCCCCCAGAACAAGAAG	AGAACGACACCTGGGCAAGACGAT	441	Conventional RT-PCR
Id2(exogenous)	Inhibitor of DNA binding 2		AGCATCCCCCAGAACAAGAAG	CCTTATTCCAAGCGGCTTCG	357	Conventional RT-PCR
Actb	actin, beta	NM_007393	CCTAAGGCCAACCGTGAAAAG	TCTTCATGGTGCTAGGAGCCA	646	Conventional RT-PCR
Cdx1	caudal type homeobox 1	NM_009880	GGACGCCCTACGAATGGAT	CCCAGAAGGCCAGCATTAGT	376	Conventional RT-PCR
Cdx2	caudal type homeobox 2	NM_007673	CGATACATCACCATCAGGAGG	TGGCTCTGCGGTTCTGAAA	94	Quantitative RT-PCR
Sox2	SRY (sex determining region Y)-box 2	NM_011443	CACAACTCGGAGATCAGCAA	CTCCGGGAAGCGTGTACTTA	190	Quantitative RT-PCR
Hnf1a	hepatocyte nuclear factor 1, alpha	NM_009327	ACCACTGCATCCCTCCTATCA	ACCTCAGGCTTGTGGCTGTAT	129	Quantitative RT-PCR
Hnf4a	hepatocyte nuclear factor 4, alpha	NM_008261	GCCACAGTTTTCCACCAAGAG	AAGGAGGACGTCTGCTTCTGA	117	Quantitative RT-PCR
Isx	intestine specific homeobox	NM_027837	AGAGGACTCCAGGCAGACAA	AGCTGCTCTGTGGTGAAGGT	120	Quantitative RT-PCR
Sox21	SRY (sex determining region Y)-box 21	NM_177753	TTGCTTTGCACTTCGGAACC	AGCCCAACGTATCGTAAAGC	75	Quantitative RT-PCR
Barx1	BarH-like homeobox 1 (Barx1)	NM_007526	CGGAGTCGCACCGTATTCAC	ACGTCTTCACCTGTAACTGGCTCA	136	Quantitative RT-PCR
Pitx1	paired-like homeodomain transcription factor 1	NM_011097	TGTGGACCAACCTCACTGAAC	ACAGGTCCAACTGCTGGTTC	96	Quantitative RT-PCR
Isl1	ISL1 transcription factor, LIM/homeodomain	NM_021459	ATGATGGTGGTTTACAGGCTAAC	TCGATGCTACTTCACTGCCAG	174	Quantitative RT-PCR
Six2	sine oculis-related homeobox 2	NM_011380	CACCTCCACAAGAATGAAAGCG	CTCCGCCTCGATGTAGTGC	165	Quantitative RT-PCR
Sult1d1	sulfotransferase family 1D, member 1	NM_016771	TGTAATGAAGGAGAATCCTAGTGC	GATTCTTCCAATCGCCTGAA	110	Quantitative RT-PCR

Table S7 (*continued*)

Gene Symbol	Gene description	Genebank accession No.	Forward Primer (5' to 3')	Reverse Primer (5' to 3')	Size (bp)	Detection
Spink3	serine peptidase inhibitor, Kazal type 3	NM_009258	TTTGGCCCTGCTGAGTTTAG	AGTAATTCCGTCAGTCCCACAC	130	Quantitative RT-PCR
Anxa13	annexin A13	NM_027211	GTCTTATCCAGCAGGACATC	TGTTGCTTCGTGTGCATAGG	238	Quantitative RT-PCR
Muc13	mucin 13, epithelial transmembrane	NM_010739	TTCAGCTGATCCTCACCATC	TCATCCTCAATCAGCCTCTG	133	Quantitative RT-PCR
Fabp1	fatty acid binding protein 1, liver	NM_017399	CCAATTGCAGAGCCAGGAGA	CCCCTTGATGTCCTTCCCTTT	91	Quantitative RT-PCR
Krt15	keratin 15	NM_008469	TGTGGAGGAATCAGTGGATG	TCCCAGCCTCTATGTCCAGT	135	Quantitative RT-PCR
Foxa2	forkhead box A2	NM_010446	CCATCAGCCCCACAAAATG	CCAAGCTGCCTGGCATG	89	Quantitative RT-PCR
Cym	chymosin	NM_001111143	AGCAAGTCATTGGAGCTGTG	ATAAGCGTAGGGTGGCAGTG	128	Quantitative RT-PCR
Irx3	Iroquois related homeobox 3	NM_008393	TCGCTGTAGTGCCTTGGAAGTG	ATAAGACCAGAGCAGCGTCCAGA	101	Quantitative RT-PCR
Irx5	Iroquois related homeobox 5	NM_018826	GATCTGGAGAAGAACGACGAGGAC	CCTTCGGAGGACGACTCCTTAAA	194	Quantitative RT-PCR
Adcy8	adenylate cyclase 8	NM_009623	GCCCTGGCTGACTTCTCTCT	CACCTGCCACCACTGAGCCAT	115	Quantitative RT-PCR
Muc1	mucin 1, transmembrane	NM_013605	TACCCTACCTACCACACTCA	TGCTACTGCCATTACCTG	94	Quantitative RT-PCR
Tff2	trefoil factor 2	NM_009363	ACCCGGGCATCAGTCCCGA	GCAGCTCCCAGGGAACGGGT	152	Quantitative RT-PCR
Actb	actin, beta	NM_007393	TGACAGGATGCAGAAGGAGA	GCTGGAAGGTGGACAGTGAG	131	Quantitative RT-PCR
